# Time-critical influences of gestational diet in a seahorse model of male pregnancy

**DOI:** 10.1242/jeb.210302

**Published:** 2020-02-07

**Authors:** Francisco Otero-Ferrer, Freddy Lättekivi, James Ord, Ene Reimann, Sulev Kõks, Marisol Izquierdo, William Vincent Holt, Alireza Fazeli

**Affiliations:** 1 Grupo en Biodiversidad y Conservación, Instituto Universitario en Acuicultura Sostenible y Ecosistemas Marinos (IU-ECOAQUA), Universidad de Las Palmas de Gran Canaria, Marine Scientific and Technological Park, Carretera de Taliarte s/n, E-35214 Telde, Spain; 2 Institute of Biomedicine and Translational Medicine, Department of Pathophysiology, University of Tartu, Ravila 14b, 50411 Tartu, Estonia; 3 Perron Institute for Neurological and Translational Science, RR Block, QE II Medical Centre, 8 Verdun Street, Nedlands, WA 6009, Australia; 4 Grupo de Investigación en Acuicultura, Instituto Universitario en Acuicultura Sostenible y Ecosistemas Marinos (IU-ECOAQUA), Universidad de Las Palmas de Gran Canaria, Marine Scientific and Technological Park, Carretera de Taliarte s/n, E-35214 Telde, Spain; 5 Academic Unit of Reproductive and Developmental Medicine, Department of Oncology and Metabolism, University of Sheffield, Level 4, Jessop Wing, Tree Root Walk, Sheffield S10 2SF, UK

**Keywords:** Syngnathidae, Development, Critical windows, Gestation, Paternal effects, Nutrition

## Abstract

Sex role reversal is not uncommon in the animal kingdom but is taken to the extreme by the Syngnathidae, in which male pregnancy is one of the most astonishing idiosyncrasies. However, critical and time-dependent environmental effects on developing embryos, such as those extensively studied in mammalian pregnancy, have not been investigated in the male pregnancy context. Here, we tested the hypothesis that seahorse pregnancy is subject to ‘critical windows’ of environmental sensitivity by feeding male long-snouted seahorses (*Hippocampus reidi*) a diet deficient in polyunsaturated fatty acids during specific periods before and during pregnancy. Despite embryos being nourished principally by maternally supplied yolk, we found that offspring morphology, fatty acid composition and gene expression profiles were influenced by paternal diet in a manner that depended critically on the timing of manipulation. Specifically, reception of a diet deficient in polyunsaturated fatty acids in the days preceding pregnancy resulted in smaller newborn offspring, while the same diet administered towards the end of pregnancy resulted in substantial alterations to newborn gene expression and elongation of the snout at 10 days old. Although paternal diet did not affect 10 day survival, the observed morphological alterations in some cases could have important fitness consequences in the face of natural selective pressures such as predation and food availability. Our results demonstrate that, under male pregnancy, fine-scale temporal variation in parental diet quality and subsequent critical window effects should not be overlooked as determinants of developing offspring fitness.

## Abstract (precis)

**Summary:** Food quality has a time-dependent impact on the offspring of male seahorses, revealing new insights into male pregnancy and its potential adaptive importance for syngnathid offspring.

## INTRODUCTION

No embryo develops independently of its environment. From the onset of the developmental process, the embryo is subject to environmental fluctuations that may prompt responses which in turn leave a long-lasting imprint on the developed organism. This general hypothesis, known as the predictive adaptive response hypothesis (Bateson et al., 2014), has its origins in epidemiology, notably observations that human exposure to famine conditions (e.g. the Dutch Hunger Winter 1944–1945; Heijmans et al., 2008) early in gestation led to metabolic syndromes in developed adults. The combination of adaptive fine-tuning of metabolic programmes early in development and a mis-matched postnatal environment has subsequently been blamed for the onset of disease in later life. The observation that disease susceptibility was higher for individuals exposed to adversity in early gestation led to the supposition that there are specific periods of time, or ‘critical windows’ (Mueller et al., 2015) during development that are especially important for normal phenotypic and functional differentiation. By their nature, critical windows represent periods of time when developmental processes are sensitive to environmental stressors such as endocrine disrupters (Degitz et al., 2000; Monteiro et al., 2015), hypoxia or hyperoxia (Bavis, 2005; Dzialowski et al., 2002), or reduced nutrient availability (MacLaughlin and McMillen, 2007). As well as epidemiological studies, evidence for both the predictive adaptive response hypothesis and critical windows have emerged from detailed experimental studies on rodents (Fleming et al., 2012; Watkins et al., 2008). For instance, maternal low protein diet (LPD) imposed during the pre-implantation period induces adverse growth, metabolic and behavioural outcomes in subsequent offspring, but the same outcomes were not observed when LPD was sustained throughout pregnancy (Watkins et al., 2008). This suggests that adaptive responses are sufficient to protect offspring fitness when conditions are consistent but become maladaptive in the absence of such consistency.

While critical windows of developmental sensitivity are not restricted to pregnancy or embryonic development, the mammalian pregnancy affords the possibility for intimate communication between parent and offspring during embryonic development and potentially frequent maternal adjustment of care, thus providing the mother with enhanced opportunities to fine-tune offspring development to match a predicted postnatal environment. Although viviparity (live-bearing) has been widely adopted across more than 150 vertebrate lineages including mammals, squamates and fish, in nearly all cases, only the females become pregnant. However, an evolutionary novelty (Wagner and Lynch, 2010) has equipped male seahorses and their syngnathid relatives, the pipefish, with a brood pouch that allows them to become pregnant (Stölting and Wilson, 2007). In seahorses, the oocytes are fertilised as they enter the brood pouch, and the resultant embryos become embedded within well-vascularised depressions in the interior pouch lining (Carcupino et al., 2002; Stölting and Wilson, 2007) where they develop until hatching. The intense vascularisation of the interior pouch lining and intimate contact of the embryos therewith implies that the brood pouch is far more than simply a protective container for embryos. Indeed, morphological and molecular research has established that the seahorse brood pouch displays many functional attributes that resemble the mammalian uterus (Whittington et al., 2015). Having evolved in such an unconventional context, the seahorse pouch offers a unique opportunity to investigate the functional importance of interactions between embryos and the parental environment in a pregnancy context that is uniquely independent of maternal physiology. Specifically, the question of whether male pregnancy allows for a similar degree of fine-tuning of embryonic development to mammalian pregnancy has been relatively unexplored.

Syngnathid oocytes provide developing embryos with maternal nutrients sequestered in the yolk, and several studies have established maternal body size and quality of the female diet as important correlates of reproductive success, egg size and offspring fitness (Braga Goncalves et al., 2011; Otero-Ferrer et al., 2016; Planas et al., 2010; Saavedra et al., 2014; Vincent and Giles, 2003). By contrast, the male environment was once thought not to make a significant contribution to offspring fitness. However, radio-labelling experiments on pipefish, which possess a brood pouch functionally similar to that of seahorses, have shown that syngnathid embryos are able to receive nutrients from the male diet, via the brood pouch (Kvarnemo et al., 2011), suggesting that paternally supplied nutrition may have been overlooked as a contributor to offspring fitness. Concordantly, Sagebakken et al. (2017) showed that survival of pipefish embryos following immune challenge increases with the brooding male's nutritional state, suggesting that paternal nutrient provisioning can promote survival (Sagebakken et al., 2017). Similarly, our previous study on seahorses (Otero-Ferrer et al., 2016) showed that males fed a standard commercial aquarium diet with a lower proportion of polyunsaturated fatty acids (PUFAs) before and during pregnancy produced unusually large offspring with poorer survival compared with the offspring of males fed a wild-caught diet with higher PUFA content. PUFAs, which include eicosapentaenoic acid (EPA), arachidonic acid (ARA) and docosahexaenoic acid (DHA), are essential for healthy development and growth of embryos as they constitute key substrates for the production of eicosanoid signalling molecules, which have key regulatory functions relating to immunity and cell growth (Pittman et al., 2013). The commercial diet, given its lower content of essential PUFAs, was likely to have been restrictive to seahorse pouch function and/or normal embryonic development compared with the wild-caught diet with its higher PUFA content, thus leading to the deleterious effects observed in the offspring of commercial diet-fed males.

Here, we used the long-snouted seahorse (*Hippocampus reidi*) as a model of male pregnancy to ask whether male pregnancy is subject to critical windows similar to those observed previously in mammals. We expanded on our previous work on the effects of a low-PUFA diet (Otero-Ferrer et al., 2016), and hypothesised that the effects of paternal commercial diet on offspring would differ depending on the time window during which the commercial diet is encountered, as the degree of developmental plasticity is likely to differ according to the stage of embryonic development. We tested this hypothesis by experimentally manipulating the adult male's diet by providing males with the commercial diet, instead of wild-caught diet, at different stages of the reproductive process, for specific periods either before or during pregnancy. Subsequently, we measured offspring phenotypes such as morphology, fatty acid composition and survival. Furthermore, we applied RNA sequencing to examine whether paternal diet induces alterations to gene expression profiles at birth and whether these depend on the timing of manipulation. Specifically, we hypothesised that the commercial diet imposed before or throughout pregnancy would be restrictive to offspring growth as a result of reduced nutrient provisioning and/or impairments to pouch function, but that exposure sustained throughout pregnancy from an early stage would enable offspring to adapt such that normal developmental patterns could be maintained. We expected that some degree of growth distortion or physiological dysregulation may result from imposing the commercial diet around the time of conception (periconception) because of the heightened sensitivity of early embryos to long-term inducible changes, as observed in mammals (Fleming et al., 2015; Watkins et al., 2008), although seahorse embryos may be less susceptible than mammals in this regard because of their abundance of available yolk resources during this early period. Finally, we expected embryos exposed to paternal commercial diet at the end of pregnancy would be especially sensitive given that they would be less reliant on maternally derived nutrients, but that adaptive responses may not manifest until later in postnatal development.

## MATERIALS AND METHODS

### Animal source and husbandry

The experiments reported here were conducted with the approval of the AquaExcel ethics committee and the ethics committee of the University of Las Palmas de Gran Canaria, Spain. The work complied with EU Directive 2010/63/EU and authorisation was covered by a REGA licence (ES350260026567). The handling practices complied with the ASAB guidelines for the treatment of animals in behavioural research and teaching (ASAB, 2001).

Twenty-one breeding pairs (males and females) of long snouted seahorses (*Hippocampus reidi* Ginsburg 1933), aged approximately 12–18 months, were randomly selected from various captive batches held at the aquaculture research facilities of the University of Las Palmas (Telde, Gran Canaria, Canary Islands, Spain). Each pair of fish was placed into a 75 l square glass aquarium supplied with flow-through ambient seawater previously purified using a 5 μm sand filter (S500, Jazzi, Guangzhou, China), biological filter (blue tower, Aqua Medic, Bissendorf, Germany), skimmer (Turboflotor 5000 Shorty Compact, Aqua Medic) and UV light (56W/UV-C, Teco, Ravenna, Italy). Temperature was maintained at 25–27°C using an aquarium chiller (TC60, Teco). Oxygen level ranged from 6.5 to 7 mg l^−1^ and salinity was established at 37 parts per thousand. Aeration was provided by an air pump located outside each tank. All aquaria were illuminated for 10 h per day by broad-spectrum fluorescent tubes (FHQ 24W reef white, 24 W, 10,000 K, Aqua Medic). Each aquarium was isolated with black PVC walls to avoid visual interaction between seahorse pairs in different tanks. Furthermore, cleaning of each tank to remove animal faeces was undertaken every day before feeding episodes. Finally, two seaweed-like plastic attachment substrata were placed into each aquarium to provide holdfast and habitat for the animals.

Prior to commencing the experimental procedures, male and female adult seahorse couples were established and randomly assigned (random number generator) to one of the five experimental groups (see ‘Experimental design’, below). Male and female body sizes (mass and standard length) were matched as closely as possible. Each adult was weighed by placing it in an individual beaker containing a known volume of water and weighing the beaker to the nearest 0.1 mg.

During an initial period of 30–45 days, when all of the seahorses were fed with wild-caught mysids, most (20/21) of the couples mated and produced offspring. The start of the initial pregnancy was determined by noting the time either when courtship and mating were observed or when the male's pouch had become swollen. Seahorse couples typically synchronise their reproductive cycles such that females are ready to transfer eggs to the male shortly after the latter has given birth, and thus courtship and re-mating can occur within a short time frame following parturition (Vincent, 1995). Concordantly, we observed that most of the seahorse pairs mated within hours of the preceding parturition event, and thus we considered the duration of the inter-brood interval to be equivalent to the duration of pregnancy. As we were not always able to pin-point the exact time of mating, we therefore used inter-brood interval as a proxy for pregnancy duration. This stage of the study established that the average duration of pregnancy was about 14 days and the information was used to predict the next expected mating dates (pregnancy calendar). The date of parturition and mating was predictable to within ±3 days when the preceding date of parturition was used as a reference point, and thus we were able to establish a feeding timetable for each individual male which corresponded with the timing of a given pregnancy.

We allowed couples to produce multiple successive broods before beginning the experimental dietary manipulations. This was necessary as two of the experimental treatments required us to manipulate the diet during one pregnancy and examine the effects on the outcome of the next pregnancy (before conception and periconception treatments, see ‘Experimental design’, below). Although, for a given couple, the exact pregnancy that was subject to dietary manipulation varied (between the second and seventh pregnancy; see Table S1), the dietary manipulations and collection of corresponding broods for all couples were carried out within the same 11 week period.

### Diets

Each adult seahorse was fed daily with one of two mysid regimes (wild caught or commercial) at a daily ratio of 10% of its body mass. Although seahorse couples were housed in individual tanks, males and females were fed separately by hand with either wild-caught or commercial diet according to the requirements of the experimental design. Netting was used within the tanks to separate the males and females during feeding periods and to prevent cross-contamination of feed during the experiment. Uneaten food was removed to avoid mixing of the diets between the pair.

The commercial diet consisted of frozen mysids (*Neomysis* sp.); it is used widely as a diet for marine fish in aquaria (Ocean Nutrition, Essen, Belgium); details of the lipid and fatty acid analysis have been published previously (Otero-Ferrer et al., 2016). Wild diet consisted of various mysid species (*Leptomysis* sp. and *Paramysis* sp.) collected with a 500 μm hand net in the wild (northeast shallow coast of Gran Canaria). These were transported to the breeding facilities using polyvinyl chloride (PVC) containers, frozen and stored at −80°C until the beginning of the feeding experiment.

Food was distributed between two equal-sized meals (at 09:00 h and 14:00 h) 6 days per week. Food administration rate was not changed during the period of the experiment except that halfway through the study period the males and females were weighed so that the food/mass ratio could be recalculated and adjusted. During the feeding period, all tanks remained without an in-flow of fresh seawater for 1 h.

Females received wild-caught diet throughout the course of the experiment, but the feeding regimes administered to males were varied by providing commercial diet for specific periods before, during or after mating as described below (‘Experimental design’).

### Experimental design

**Fig. 1.**
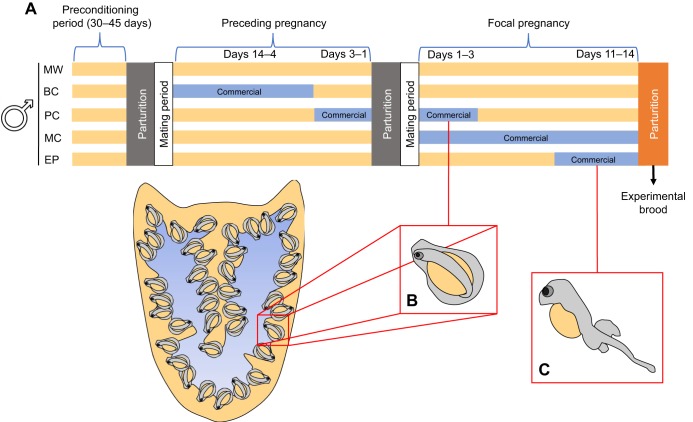
**Representative diagram of the experimental framework.** (A) Simplified time line of dietary manipulations showing the type of diet (wild or commercial) administered to males of each experimental group during specific experimental periods. Yellow bars represent periods during which males were fed the wild diet, while blue bars denote periods during which males in specific groups were administered the commercial diet. The parturition of the experimental brood is shown in orange, while previous parturition events (for which broods were not examined) are shown in grey. The pregnancy corresponding with the production of the experimental brood is referred to as the focal pregnancy. MW, male wild; BC, before conception; PC, periconception; MC, male commercial; EP, end pregnancy. (B) Representative depiction of a seahorse embryo as it would appear in the brood pouch during the first few days of pregnancy. (C) Representative depiction of a well-developed seahorse embryo (yolk sac larva) as it would appear in the brood pouch during the last few days of pregnancy. Embryo drawings adapted from Novelli et al. (2018).

To investigate whether a change in paternal diet during hypothesised critical windows induces changes in the offspring, five different experimental feeding regimes were provided to the adult males. The male wild (MW, *N*=5) group served as a control in which all males were fed the wild diet throughout the experiment. The four other groups received the commercial diet during a specific time period before the production of a given brood, such that influences of paternal dietary manipulation on characteristics of those broods could be examined. These four groups were designated BC (before conception, *N*=4), PC (periconception, *N*=4), MC (male commercial, *N*=3) and EP (end pregnancy, *N*=4), respectively. Please refer to Fig. 1 for a simplified representation of the timing of dietary manipulations for each treatment group in relation to the production of experimental broods.

BC males received commercial diet from the day of one parturition/mating event until approximately 3 days before the next predicted parturition/mating event, after which they received wild-caught mysids (average duration of commercial diet: 9.25 days). Offspring from the next parturition were thus examined for changes in response to dietary manipulation during the previous pregnancy (i.e. before conception of the examined brood).

PC males received the commercial diet beginning from 3 days before the predicted time of a given parturition/mating event until 3 days after this event, from which point they received wild diet (average duration of commercial diet: 6.75 days). The brood produced in the next parturition event was thus examined for changes in response to dietary manipulation around the time of its conception.

MC males received wild-caught mysids prior to a given parturition/mating event and were then fed with commercial diet for the entire duration of the subsequent pregnancy (average duration of commercial diet: 13 days). The subsequent brood was thus examined for changes in response to dietary manipulation during gestation, i.e. from the early embryo stage (Fig. 1B) until parturition.

Finally, EP males received wild-caught mysids up until 6 days before a given parturition event was predicted, i.e. the last six days of a pregnancy, from which point they were fed the commercial diet until parturition (average duration of commercial diet: 3.75 days). The subsequent brood was thus examined for changes in response to dietary manipulation at the end of gestation. As embryos hatch from their chorions during the last few days of pregnancy (Novelli et al., 2018), the offspring would have been free-swimming ‘yolk sac larvae’ during this period of manipulation (Fig. 1C).

We began with 21 breeding pairs in total (5 in the MW group and 4 in all other groups), although one MC pair failed to produce a brood, such that the number of replicate broods per group ranged from 3 to 5.

Following parturition of experimental broods, the number of offspring was recorded, and different phenotypic traits were measured at day 0 (newborn) and day 10. Between 80 and 120 newborns were kept for rearing to day 10 and remaining newborns were killed for mass measurement, fatty acid analysis or RNA sequencing. Brood sizes for each seahorse couple can be found in Table S1.

Newborns derived from previous parturition events were not subsequently used for this study and were therefore humanely killed by anaesthetic overdose (clove oil, 50 ppm in seawater).

### Assessment of offspring dimensions, mass and survival

Linear dimensions of newborn and 10 day old seahorse offspring were measured using a profile projector (Mitutoyo PJ-A3000, Kawasaki, Japan) under anaesthesia (clove oil, 25 ppm in seawater), as described previously (Otero-Ferrer et al., 2010). For measurement, anaesthetised newborns were transferred to a glass slide using a Pasteur pipette and were killed by anaesthetic overdose (clove oil, 50 ppm in seawater) after measurements were complete. The dimensions measured were height (Ht), snout length (SnL), head length [HL, distance from the tip of the snout (upper jaw) to the mid-point of the cleithral ring], trunk length (TrL, straight-line distance from the mid-point of the cleithral ring to the mid-point of the last trunk ring) and tail length (TL, distance from the mid-point of the last trunk ring to the tip of the outstretched tail) as defined by Lourie et al. (2004). Tails were measured in segments to account for curvature. Standard length (StL) was calculated as the sum of HL, TrL and TL. These linear dimensions were also used to calculate size ratios, specifically snout length to standard length (SnL/StL), snout length to head length (SnL/HL) and trunk length to tail length (TrL/TL).

In most cases, 10 randomly selected newborn seahorses (day 0) from all males were measured, as well as 6 randomly selected 10 day old offspring from each of 3 MW, 2 BC, 4 PC, 2 MC and 3 EP males, with limited broods available for BC and MC as a result of mortality. Total sample sizes for offspring size measurements are summarised in Table S2. Experimenters were not blinded as to group allocations.

For the assessment of dry mass, 15 euthanised offspring per brood (including those used for measurement of linear dimensions) were separated into three batches of 5 which were placed into an oven (Jouan, EU 280 EL TS SN Inox, Saint-Herblain, France) at 105°C until the mass became constant. Because of their small size, offspring were weighed together in each batch of five and the measurement divided by 5 to obtain an average (thus we obtained averages from three batches from each brood). The mass measurements were taken with a precision balance (Mettler Toledo, AG204, Greifensee, Switzerland), which was accurate to the nearest 0.1 mg.

Survival of newborns from the experimental broods was estimated by dividing 80 or 120 (depending on brood size) randomly selected newborns produced by each couple into two or three 40 l tanks (40 offspring per ‘sub-brood’ to control for effects of population density) and making daily counts of the number surviving. Newborns were fed twice daily with enriched *Artemia* nauplii, following a previously established protocol (Garcia-Manchón et al., 2013). Owing to space constraints, the survival tests had to be terminated at day 10.

### Fatty acid determination analyses

Groups of approximately 100 newborns from each brood were washed twice with distilled water and freeze dried (−80°C) for subsequent analysis. Crude lipids from newborn seahorses were extracted using the method described by Folch et al. (1957). The lipid extracts were divided into three portions for subsequent triplicate analyses. Fatty acid methyl esters from total lipids were prepared by transmethylation (Christie, 1982) then separated and quantified by gas chromatography using conditions described previously (Izquierdo et al., 1990).

### Statistical analysis of phenotype data

Statistical analyses were carried out using ‘R’ version 3.5.1 (http://www.R-project.org/).

The effects of treatment on brood size (number of offspring born per unit maternal body mass) were analysed using analysis of covariance (ANCOVA) with paternal mass as an additional covariate. Duration of the inter-brood interval was also analysed by ANCOVA, using both paternal and maternal mass as covariates.

To evaluate the effects of treatment on offspring mass, linear dimensions and size ratios, we used linear mixed effects models fitted by residual maximum likelihood (REML) using the ‘lme4’ package (Bates et al., 2015). Because multiple offspring were measured per replicate brood (or three pools of 5 offspring per brood, in the case of newborn mass), ‘brood’ was included as a random effect term in the models, which also accounted for genetic influences on size traits. *F*-tests with Kenward–Roger approximation of degrees of freedom (‘lmerTest’ and ‘pbkrtest’ packages; Halekoh and Hojsgaard, 2014; Kuznetsova et al., 2017) were used to assess statistical significance of paternal treatment, and *post hoc* tests with Dunnett correction for multiple comparisons (‘emmeans’ package; https://CRAN.R-project.org/package=emmeans) were used to examine differences between the control group (MW) and each of the other groups. As newborn dimensions and size ratios exhibited skewed distributions, we applied the ‘Gaussianisation’ algorithm from the ‘LambertW’ package (https://CRAN.R-project.org/package=LambertW) to normalise the distributions of model residuals. Day 10 offspring size data were normally distributed and thus not subject to transformation.

To help understand the multivariate outcomes of the various treatments on morphological traits, data were subjected to K-means cluster analysis. All of the newborn offspring were combined into a single group without regard to treatment. We used the variables StL and HL, the data values were standardised (mean=0 and s.d.=1), and we specified two clusters (termed ‘large’ and ‘small’ for clarity). Counts of individuals classified as large and small were further compared statistically between treatment groups using a generalised linear model followed by Tukey tests.

As offspring fatty acid data were not normally distributed but did not require a mixed model approach (only one value per fatty acid per brood), Kruskal–Wallis tests were used to evaluate the effect of treatment on offspring fatty acid levels and ratios, followed by Dunn's multiple comparison tests with Bonferroni adjustment (R package ‘PMCMR’; https://CRAN.R-project.org/package=PMCMR) to compare levels and ratios in the MW group with those of each of the other treatment groups.

To evaluate 10 day survival, for which three sub-broods were examined for each replicate brood, we used a generalised linear mixed model (glmer function in lme4) with binomial error distribution, as appropriate for count data, with ‘brood’ included as a random effect term.

### RNA extraction and sequencing

For whole-transcriptome analysis, newborn offspring were collected from 4, 3, 4, 3 and 4 broods from the MW, BC, PC, MC and EP treatments, respectively. Total RNA was extracted from newborn offspring stored in RNAlater (Life Technologies Ltd, Paisley, UK). Briefly, pooled whole-body samples (pools of 5–8 per brood) were homogenised using a Fastprep-24 instrument with lysing matrix D tubes (MP Biomedicals, Santa Ana, CA, USA). For total RNA extraction, the mirVana miRNA Isolation Kit (Thermo Fisher Scientific, Waltham, MA, USA) was applied according to the manufacturer's protocol, but withholding the miRNA enrichment step. DNase treatment was conducted on-column during RNA purification, applying the RNase-Free DNase Set (Qiagen, Hilden, Germany). The quantity of the extracted RNA was measured using the Qubit 2.0. fluorometer and Qubit RNA HS Assay Kit (Thermo Fisher Scientific). For evaluating RNA integrity, an Agilent 2100 Bioanalyzer and RNA 6000 Nano kit (Agilent Technologies, Santa Clara, CA, USA) was used. The RNA integrity number (RIN) of all samples was ≥8.

A 50 ng sample of each total RNA was amplified with Ovation RNA-Seq System V2 Kit (NuGen Technologies Inc., Redwood City, CA, USA) resulting in double stranded DNA. This was followed by preparing SOLiD 5500 Wildfire System (Thermo Fisher Scientific) DNA fragment libraries according to manufacturers' protocols. The sequencing step was performed with the SOLiD 5500xl Wildfire platform using 50 bp single-end fragment sequencing chemistry. Sequencing yielded on average 25.2 million reads per sample.

### Read alignment and expression quantification

A reference transcriptome was constructed from the predicted *Hippocampus comes* transcript sequences deposited in the NCBI RefSeq database under the NCBI BioProject PRJNA359802. These sequences were composed of both coding and non-coding transcripts, totalling 47,028 isoforms from 24,897 genes. The sequenced colour-space reads were aligned to the constructed reference transcriptome using LifeScope software (Life Technologies) mapping module with recommended settings, designed for colour-space read alignment and analysis. Prior to alignment, the reads were filtered for rRNA, tRNA and SOLiD adaptor sequences. As all gene isoforms were present in the constructed reference sequence, multiple alignment was allowed and therefore no mapping quality (MAPQ) cut-off was set. Alignments were, however, filtered on the basis of aligned bases, as only alignments with 45 consecutively aligned bases were kept; single nucleotide polymorphisms (SNPs) were allowed.

On average, 85.1% of the reads had an average per-base Phred-scale quality score greater than 20 (QV>20); 19.7% of reads mapped to the reference constructed from *H. comes* transcript sequences, and after filtering based on the number of aligned bases, 14.1% of the reads were considered for further analysis. The alignments were quantified at the gene level for subsequent differential expression (DE) analysis.

Even though the reference transcriptome and the sequenced reads are from a different *Hippocampus* species, the fraction of aligned reads was unexpectedly small. To assess whether this low level of read mapping could be attributed to contamination, the amount of RNA originating from prokaryotes and other accompanying organisms was assessed by mapping the small fraction of rRNA reads to ribosome large subunit (LSU) sequences from the Rfam database (Griffiths-Jones et al., 2003). MAPQ cut-off was set at 20 for these alignments. Although this strategy was affected by high similarity and the completeness of individual rRNA reference sequences, it was applicable to gain insight into possible contamination. The majority of the reads were observed to align to LSU sequences of species within the vertebrate sub-phylum, leading to the conclusion that the majority of the sequenced reads were of seahorse origin and the abundance of secondary organisms (parasites, pathogens and symbionts) was unlikely to be the cause of the low alignment rate.

### DE and pathway enrichment analysis

DE analysis was conducted using the edgeR package (http://bioinf.wehi.edu.au/edgeR) in R version 3.3.1 (http://www.R-project.org/). For excluding genes expressed at a low level, at least two samples in every group were required to pass a counts per million (CPM) threshold of 0.2 for a specific gene. A CPM of 0.2 translates into five reads mapped per gene given the average library size. The generalised linear model approach was used to detect DE genes in all the groups compared with the MW group. DE analysis results were visualised using pheatmap (https://CRAN.R-project.org/package=pheatmap), ggbiplot (http://github.com/vqv/ggbiplot) and ggplot2 (https://CRAN.R-project.org/package=ggplot2) packages in R. Correlation plots were visualized with the ggpubr package (https://CRAN.R-project.org/package=ggpubr). We performed a principal component analysis (PCA) of the standardised (*Z*-scored) CPM values of all genes that were differentially expressed (false discovery rate FDR≤0.05) in at least one treatment group compared with the MW group. We further assessed the extent to which expression changes of individual genes correlated with the timing of dietary manipulation by ordering the experimental groups based on the commencement of the manipulation relative to the pregnancy and calculated Spearman's rank correlation coefficient (*r*) between the described order of experimental groups and expression values of genes. The order of experimental groups based on the commencement of the manipulation was MW–BC–PC–MC–EP.

At the time of analysis, the KEGG Pathway Maps (Kanehisa and Goto, 2000) did not include annotations for any of the *Hippocampus* species. Therefore, in order to conduct pathway enrichment analysis based on KEGG Pathway Maps, the zebrafish (*Danio rerio*) annotations were adopted by matching the *H. comes* transcript sequences from the NCBI Nucleotide database with zebrafish protein sequences from the NCBI RefSeq database using blastx from NCBI BLAST^®^ version 2.2.30 (Altschul et al., 1990). Non-default parameters of the blastx command were as follows: outfmt, 6; evalue, 0.00001; max_target_seqs, 1; max_hsps, 1; word_size, 4; threshold, 18. The longest *H. comes* transcript for every gene was used in the blastx alignment as the representative sequence of the corresponding gene. This resulted in annotation of the *H. comes* genes that had remained relevant after DE analysis with corresponding zebrafish protein sequence RefSeq identifiers.

The protein sequence RefSeq identifiers were subsequently replaced with Entrez identifiers for the corresponding genes using an in-house script and NCBI RefSeq *D. rerio* entries. The DE genes annotated with *D. rerio* Entrez gene identifiers were used in the pathway enrichment analysis applying the gage() function from the gage package (Luo et al., 2009) in R. In order to obtain more power to detect potentially relevant pathways, the FDR cut-off for DE genes was relaxed from 0.05 to 0.1, allowing more genes to be included in the analysis at the risk of increasing the chance of false-positive results. Only genes differentially expressed between the EP and MW groups and corresponding fold-change values were used in the enrichment analysis. KEGG Pathway maps and DE genes were visualized using the pathview package (http://r-forge.r-project.org/projects/pathview/).

## RESULTS

### Outcome of pregnancy and newborn morphology

None of the treatments affected brood size (number offspring per unit female body mass) relative to MW (ANCOVA, *F*_4_=1.13, *P*=0.38). However, there was a significant effect of treatment on inter-brood interval, which we took to be a proxy for pregnancy length (ANCOVA, *F*_4_=3.71, *P*=0.03). The mean inter-brood interval for the MW group was 14.8±0.39 days. This was significantly shortened in the MC group by 1.8±0.6 days (Dunnett's test, *T*_13_=2.93, *P*=0.04). The BC and EP groups showed similarly shortened inter-brood intervals although the differences were not significant (BC: *T*_13_=2.46, *P*=0.09; EP: *T*_13_=2.6, *P*=0.07), while there was no effect of PC (*T*_13_=1.36, *P*=0.48).

**Fig. 2.**
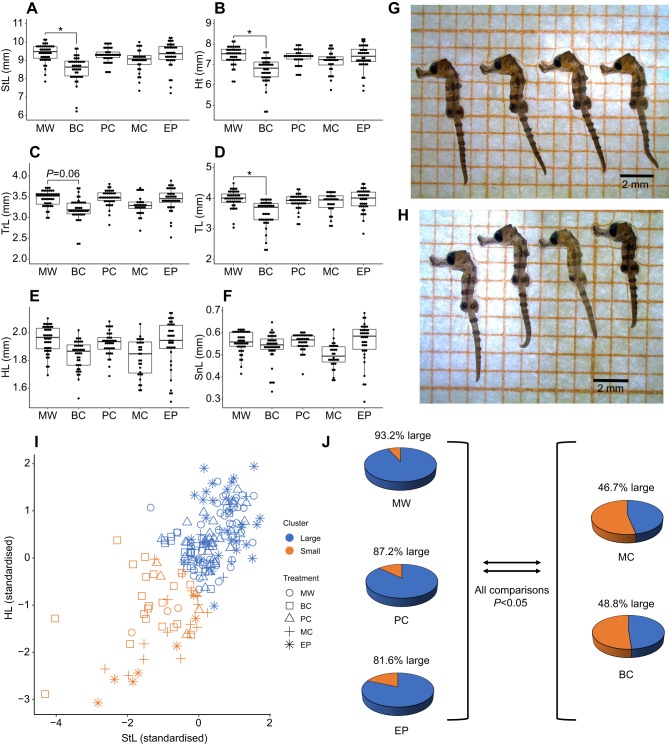
**Size traits of newborn *Hippocampusreidi* offspring derived from the five paternal dietary groups.** (A–F) Linear dimensions of newborns: StL, standard length; Ht, height; TrL, trunk length; TL, tail length; HL, head length; and SnL, snout length. Each point represents a measurement from an individual offspring (*n*=44, 41, 39, 30 and 38 total offspring from 5 MW, 4 BC, 4 PC, 3 MC and 4 EP broods, respectively). *P*-values were derived from Dunnett's multiple comparison test following linear mixed model (*P<0.05). (G,H) Representative images of newborns from the MW and BC groups, respectively. (I,J) K-means cluster analysis results. (I) Snout length (StL) and head length (HL) were standardised (mean=0) and entered as variables into a K-means cluster analysis. (J) Pie charts showing the percentage of newborns in each of the five groups that were classed as ‘large’ and ‘small’ following K-means cluster analysis. *P*-values were derived from Tukey multiple comparison test following generalised linear model. The results presented derive from a single experiment.

There were no significant treatment effects on the body mass of newborns (*F*_4,13_=0.44, *P*=0.78). Paternal diet had prominent effects on the linear dimensions of newborns if manipulated before conception (BC), but no significant differences were observed in any of the other groups compared with the MW group (Fig. 2A–F). BC newborns were significantly smaller than MW newborns with regard to StL (Dunnett's test, *T*_15_=3.26, *P*=0.02), Ht (*T*_15_=3.1, *P*=0.03) and TL (*T*_15_=3.16, *P*=0.02), and showed a trend towards smaller TrL, though this was marginally non-significant (*T*_15_=2.67, *P*=0.057).

While BC offspring exhibited significant reductions in some size traits, there was no discernible effect on SnL. This disparity is likely to explain the visible distortion of the body shape (Fig. 2G,H). Concordantly, there was a trend towards higher SnL/StL ratios in BC offspring, although there was no significant treatment effect on this ratio (*F*_4,14.9_=2.14, *P*=0.12). The SnL/HL and TrL/TL ratios were similarly unaffected (SnL/HL: *F*_4,14.8_=1.57, *P*=0.23; TrL/TL: *F*_4,14.8_=1.69, *P*=0.2).

There were no significant treatment effects on the within-brood heterogeneity of any of the linear dimensions or size ratios as determined by the intra-brood coefficients of variation (Kruskal–Wallis tests, all *P*>0.05).

Offspring from all experimental broods were clustered into two groups using K-means clustering based on Euclidean distance (Fig. 2I) and the subsequent comparisons of linear dimensions between the two clusters indicated that clusters were defined by body size; for clarity these groups were termed ‘large’ and ‘small’, respectively. The cluster identity of each individual newborn was recorded and the dataset analysed further by calculating the relative proportions (%) of large and small individuals belonging to each treatment group. Two groups of treatments could be recognized on the basis of relative proportions of large and small newborns. The MW, PC and EP treatments all resulted in the birth of >80% ‘large’ newborns (cluster 1), while MC and BC treatments resulted in a lower proportion (approximately 50%) of the ‘large’ newborns (Fig. 2J). Tukey tests following a generalised linear model of the counts of large and small offspring revealed that the BC and MC groups contained significantly lower proportions of large offspring than did the MW, PC and EP groups (all comparisons, *P*<0.05).

We did not detect significant correlations between inter-brood interval duration and most newborn size traits (brood-level means of linear dimensions or % ‘large’ offspring), although inter-brood interval showed a weak correlation with mean TrL (Spearman's rank, ρ=0.44, *P*=0.05).

### Offspring fatty acid levels and ratios

**Fig. 3.**
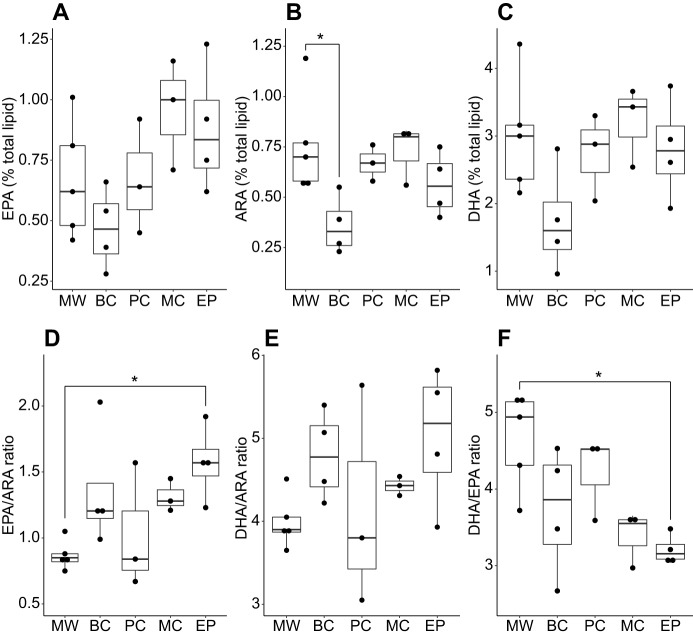
**Polyunsaturated fatty acid (PUFA) levels and their ratios in newborn *H. reidi* from the five paternal dietary groups, derived using gas chromatography.** (A–C) Levels of eicosapentaenoic acid (EPA), arachidonic acid (ARA) and docosahexaenoic acid (DHA), respectively, expressed as a percentage of total lipid content. (D–F) Ratios of EPA to ARA, DHA to ARA, and DHA to EPA, respectively. Each point represents a measurement derived from a pool of approximately 100 newborns. One such pool was derived from each of 5 MW, 4 BC, 3 PC, 3 MC and 4 EP broods. *P<0.05 Dunn's multiple comparison test following Kruskal–Wallis test. The results presented derive from a single experiment.

To assess whether paternal diet affected the composition of PUFAs in the offspring, we measured levels of the essential fatty acids EPA, ARA and DHA (% of total lipid), and their ratios (Fig. 3). Paternal treatment did not affect EPA levels (Kruskal–Wallis test, χ^2^_4_=7.4, *P*=0.12; Fig. 3A), but there was a significant effect of treatment on ARA levels (χ^2^_4_=10, *P*=0.04; Fig. 3B), which was driven by significantly lower ARA in BC newborns compared with MW newborns (Dunn's multiple comparison test, *P*=0.03). Paternal treatment did not affect DHA levels (χ^2^_4_=6.4, *P*=0.17; Fig. 3C). Paternal treatment also affected EPA/ARA ratio (χ^2^_4_=9.8, *P*=0.04; Fig. 3D), driven by significantly higher values in the EP offspring (*P*=0.02). There was no effect on DHA/ARA ratio (χ^2^_4_=6.1, *P*=0.2; Fig. 3E), but there was a significant effect on DHA/EPA ratio (χ^2^_4_=10.6, *P*=0.03; Fig. 3F), driven by significantly lower values in EP offspring (*P*=0.014).

### Survival and morphology at 10 days

**Fig. 4.**
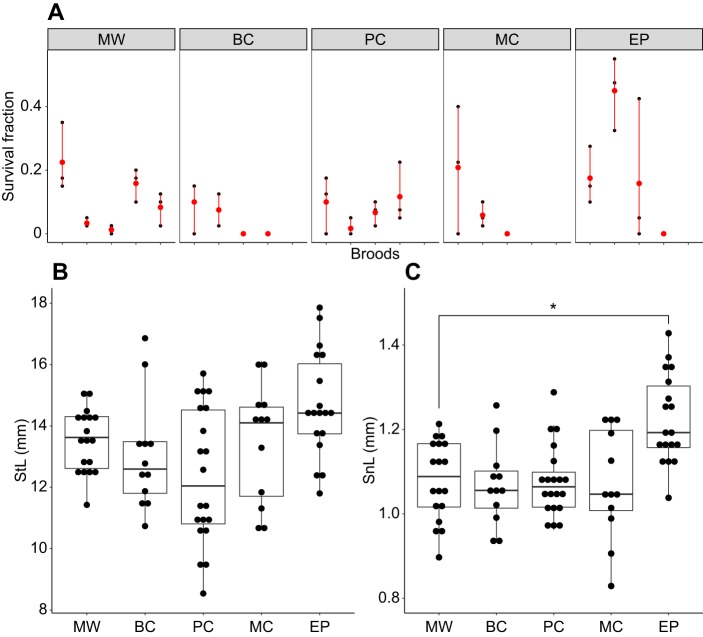
**Survival** **rates and morphological traits of 10** **day old *H. reidi* offspring from** **the** **five paternal diet groups**. (A) Fraction of offspring that survived after 10 days in each of the five experimental groups. Two to three sets of 40 offspring (sub-broods) were monitored from each brood, with black dots representing the survival fractions of sub-broods and red dots indicating the mean survival fractions of the broods. (B,C) Standard length (StL) and snout length (SnL), respectively, of 10 day old offspring. Each point represents a measurement taken from an individual offspring (*n*=18, 12, 20, 12 and 18 total offspring from 3 MW, 2 BC, 4 PC, 2 MC and 3 EP broods, respectively). Dunnett's multiple comparison test following linear mixed model (*P<0.05). The results presented derive from a single experiment.

Survival rates for all broods and sub-broods are shown in Fig. 4A. Although there was a trend towards higher survival rates in the EP group (19.6%) and lower in the BC group (4.5%) compared with the MW group (10.9%), with 2/4 BC broods exhibiting total mortality, a generalised linear mixed model with binomial error distribution revealed no significant effect of treatment on 10 day survival (full versus null model, χ^2^_4_=3.23, *P*=0.52).

In regard to morphological traits on day 10, StL was not affected by paternal diet (*F*_4,8.8_=1.21, *P*=0.37; Fig. 4B), and the only statistically significant treatment effect to emerge concerned SnL (*F*_4,8.4_=6.7; *P*=0.01). This difference was driven by the EP group in which snouts were approximately 0.15±0.04 mm longer than in the MW group (Dunnett test, *T*_8.2_=3.9, *P*=0.014) (Fig. 4C). Unfortunately, because of brood mortality, we were limited only to two broods from each of the BC and MC treatments for day 10 size measurements, meaning we were unable to reliably assess the effects of these specific treatments.

### Gene expression profiles in newborn offspring

**Fig. 5.**
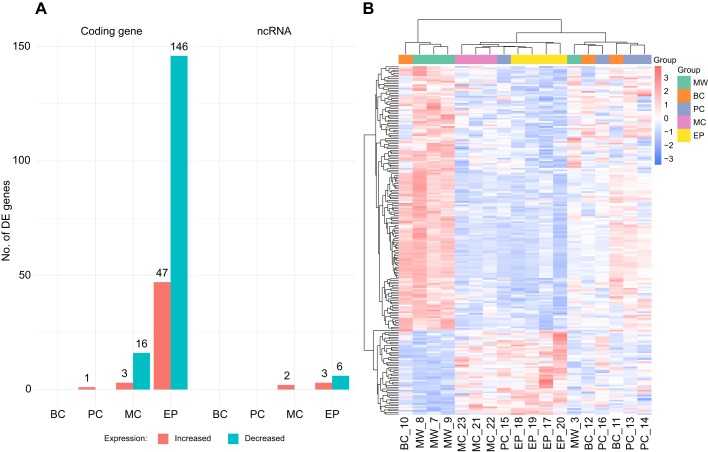
**Differentially expressed genes in the offspring of** **the** **experimental groups.** (A) Bar plots represent the number of genes differentially expressed (DE) at a false discovery rate (FDR)≤0.05 in the offspring of all experimental groups. The DE genes are further separated by whether they are upregulated (red) or downregulated (blue) in the experimental group compared with the MW group and by the type of gene: coding or non-coding (ncRNA). (B) Expression levels of genes differentially expressed at FDR≤0.05 in at least one of the comparisons between experimental groups and the MW group. Samples and genes were clustered based on Manhattan distance calculated from *Z*-score values. The mean and standard deviation for the *Z*-score calculation were derived from count per million (CPM) values across all groups. The results presented derive from a single experiment.

Most DE genes were detected in the groups in which the diet change occurred during pregnancy, with the highest number of differences observed between the MW and EP groups: 202 DE genes (coding and non-coding) at FDR≤0.05, most of which were downregulated in the EP group (Fig. 5A). No DE genes were observed in the BC group compared with MW group at FDR≤0.05, while only one DE gene was observed in the PC group compared with the MW group: predicted alpha-2-macroglobulin-like protein 1 (RefSeq ID: XM_019861357.1; log_2_FC=3.22, FDR<0.001). Lists of DE genes in the two other groups can be found in Tables S3 and S4. Intergroup differences were clearly visible in the gene expression profiles of the seahorse offspring based on the unsupervised hierarchical clustering of *Z*-score values based on Manhattan distance (Fig. 5B). This most importantly emphasises the expression profile differences between the MW group and the MC and EP groups, which, with the exception of one MW sample, all formed distinct clusters.

**Fig. 6.**
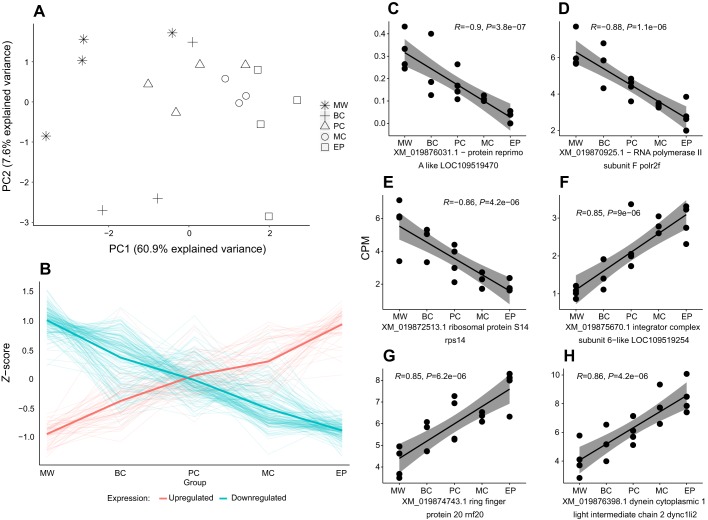
**Correlations between the degree of differential expression and time point of diet change in the course of pregnancy.** (A) Plot of the first two components of a principal component analysis (PCA) based on CPM values of genes differentially expressed at FDR≤0.05 in at least one of the comparisons between the MW group and all other groups. (B) The time-dependent relationships between the dietary manipulation and the normalised expression values (*Z*-score of CPM) of DE genes. Faded lines show the mean expression level of each gene differentially expressed at FDR≤0.05 in at least one of the treatment groups compared with the MW group (red for upregulation and blue for downregulation in any experimental group compared with the MW group). The bold red and blue lines represent the mean expression values of all upregulated and downregulated genes, respectively. (C–E) Three genes (XM_019876031.1, XM_019870925.1, XM_019872513.1) with the largest negative correlation coefficient (*r*) values with *P*-values not corrected for multiple testing. The experimental groups were interpolated as numerical values as follows: MW – 1, BC – 2, PC – 3, MC – 4, EP – 5. Spearman's rank correlation test was used. (F–H) Three genes (XM_019875670.1, XM_019874743.1, XM_019876398.1) with the largest positive correlation coefficient (*r*) values with *P*-values not corrected for multiple testing. The results presented derive from a single experiment.

A principal component analysis (PCA) revealed that the degree of sample segregation away from the MW group on PC1 was greater for the MC and EP groups, for which the manipulation was initiated or sustained later in the reproductive process (Fig. 6A). Concordantly, the expression levels of most DE genes displayed a directional trend across experimental groups, whereby the differences in normalised expression values (*Z*-scores) of DE genes compared with the MW group was the strongest in the EP group and weakest in the BC group, while the *Z*-scores of both upregulated and downregulated genes in the PC group were about half-way between the respective *Z*-scores of the MW and EP groups (Fig. 6B). Thus, the extent of differential expression increased as the time of dietary manipulation approached the end of pregnancy. This could be further illustrated by coding the experimental groups as consecutive numbers representing the groups ordered by the moment of diet change from before pregnancy to the end of pregnancy and conducting a simple correlation analysis (Fig. 6C–H). This resulted in 97% of DE genes displaying a moderate correlation (*r*≥0.5) and 60% of genes displaying a strong correlation (*r*≥0.7) based on Spearman's rank correlation coefficient (*r*) values. After correcting for multiple testing using the Bonferroni correction, 28% of total correlations remained significant at α=0.05.

KEGG pathway analysis of DE genes at FDR≤0.1 in the EP group compared with the MW group resulted in one significantly downregulated pathway: the ribosomal proteins (Table S5, Fig. S1).

## DISCUSSION

In seahorses, the majority of attention has focused on egg quality in females (Planas et al., 2010; Saavedra et al., 2014; Woods, 2000), and paternal influences have hitherto been understudied. We tested the effects of carefully timed paternal dietary manipulations on seahorse pregnancy and subsequently demonstrated that reproduction in seahorses is sensitive to male food quality during certain time windows.

### Effects of dietary manipulation on inter-brood interval and brood size

The first notable influence of paternal diet was the significant shortening of the inter-brood interval (which we considered to be a proxy for pregnancy length) in the MC group (commercial diet throughout pregnancy). The result is reminiscent of observations that stress in other live-bearing fish (Evans et al., 2007) and in mammals tends to shorten pregnancy length (Vrekoussis et al., 2010), suggesting that the commercial diet treatments were mildly stressful to male seahorses. It is also reminiscent of observations of pipefish, which have a tendency to shorten brood retention if presented with a larger alternative mate in a phenomenon reminiscent of the ‘Bruce effect’ in mammals (Cunha et al., 2018), suggesting that both seahorses and pipefish have evolved mechanisms to control the duration of brood retention in response to environmental cues.

### Complex, time-dependent influences on offspring traits

The offspring phenotypic responses to paternal diet across the various treatment groups, although subtle, were complex, with different responses depending on the timing of the manipulation. For instance, the BC treatment (commercial diet before conception) was associated with significant reductions in specific size traits at day 0 (StL, Ht and TL) and reduced ARA levels, but did not induce any significant changes in gene expression. By contrast, the EP treatment (commercial diet at the end of pregnancy) did not affect size traits at day 0 but altered the DHA/EPA and EPA/ARA ratios, as well as increasing the snout length on day 10 and displaying the highest number of DE genes on day 0. Importantly, because the EP manipulation was of a shorter duration than the BC and MC treatment, the results demonstrate that parental environmental influences can depend as much, if not more strongly, on the timing as on the duration of a parental exposure. Our findings are broadly concordant with observations from a rodent model which showed that the consequences of maternal dietary restriction on offspring fitness depend critically on the time and duration of the exposure (Watkins et al., 2008). However, manipulation of the diet during the periconception period, which has been identified as a critical window in mammalian pregnancy (Watkins et al., 2008), had no effect on seahorse offspring (PC treatment). This is not surprising given that seahorse embryos, in contrast to mammals, rely almost exclusively on nutrients of maternal origin until the later stages of development. Furthermore, it suggests that embryos do not initiate predictive adaptive mechanisms in direct response to paternal nutrient availability at this early stage. Thus, the role of yolk-supplied nutrients constitutes a fundamental difference between syngnathid and mammalian pregnancies which is an important determinant of the sensitivity of pregnancy to dietary disruption during periconception. It is also possible that embryos may have been subject to stress as a result of impairments to pouch function during the periconception period, but any such impairments may have been too transient to induce substantial adaptive responses.

The significant reductions in size traits of newborns from the BC treatment may suggest that optimal pouch function hinges on the quality of diet available to males prior to mating, and that attempting to correct for poor nutrition during the subsequent pregnancy is not effective. Indeed, low ARA and EPA contents in the pouch may impair its osmoregulation or immune functions that are mediated by eicosanoids derived from these particular fatty acids (Izquierdo and Koven, 2011). In turn, unsuitable conditions in the pouch caused by the low-quality diet would impose a stress upon the developing embryos and may promote the catabolism of ARA and EPA by cycloxygenase enzymes to overcome such stress, which may have led to reduced ARA levels in the offspring (Izquierdo et al., 2000). Alternatively, size differences in BC offspring may have come about as a result of female choice. For instance, some evidence suggests that female pipefish (*Syngnathus typhle*) are able to adjust the level of protein investment in their eggs in order to compensate for a reduced perceived quality of their partner, even within a short time frame (Braga Goncalves et al., 2010). It is therefore possible that the commercial diet altered the perceived quality of BC males prior to mating and subsequently influenced female investment decisions. However, this explanation could also be deemed unlikely given the monogamous mating system of *Hippocampus* seahorses, which is less likely to incentivise such plasticity in maternal investment compared with the polygamous mating system of pipefish such as *S. typhyle*. Furthermore, evidence that females can adjust egg resource levels so close to the time of mating remains limited.

Although sustaining the commercial diet throughout pregnancy (MC treatment) did not significantly affect any of the individual linear dimensions, it produced a high proportion of newborns classified as ‘small’ by the cluster analysis using StL and HL as input variables. This suggests that the effect of restricted PUFA availability throughout pregnancy is similar to the effect of restriction before pregnancy, and thus may have been driven by similar mechanisms. It is possible that the effect of both MC and BC on the proportion of small offspring was at least partly driven by earlier parturition resulting in the release of less developed offspring (Dial et al., 2017; Ord et al., 2019), given that inter-brood interval was significantly shorter for MC males and shortened to a similar extent (albeit not significantly) for BC males. However, the duration of the inter-brood interval did not significantly correlate with most newborn size traits, and EP males also had similarly shortened inter-brood intervals (though not significant) while not showing any clear differences in newborn size traits, both suggesting that variation in pregnancy duration is insufficient to explain newborn size differences.

Despite the evidence for smaller offspring size in the BC and MC groups, and lower levels of the fatty acid ARA in the case of the BC group, survival of these offspring was not significantly affected. Although this would initially imply that smaller size does not impede their fitness, smaller body size has been shown to increase the risk of mortality from predation in pipefish (Ahnesjo, 1992), suggesting that smaller body size does indeed incur a fitness cost in a natural setting. Overall, however, the lack of influence on mortality implies that offspring were able to withstand stresses imposed by these treatments, presumably by adjusting their prenatal growth rate to the prevailing environmental conditions. This would be consistent with the thrifty phenotype hypothesis (Armitage et al., 2005; Williams et al., 2014) in that embryos would adapt to the poorer quality pouch environment (or, alternatively, lack of maternally invested nutrients in response to lower perceived male quality) and attempt to optimize their development.

Dietary manipulation in late pregnancy (EP) did not induce significant changes in the morphology of newborns, which is not surprising given that growth is a gradual process and the change in diet occurred within a short time frame prior to parturition. However, the snouts of offspring in this group were significantly elongated relative to those of the MW group on day 10, suggesting that the manipulation at the end of pregnancy induced long-term alterations to structural development. This delayed response may be reflective of epigenetic reprogramming. Furthermore, the effect of EP treatment on relative gene expression in newborns was more than 10-fold stronger than the effect of MC treatment (both compared with MW treatment), despite both paternal groups having received the commercial diet at the end of pregnancy. This disparity of outcomes between the two groups further supports the thrifty phenotype hypothesis and suggests that embryos are plastic throughout the pregnancy, as receipt of the commercial diet from an earlier stage appears to have prevented the effect observed in EP. It could be hypothesised that in the MC context, a developmental programme is initiated early which leads to smaller body size to compensate for reduced PUFA availability. By contrast, EP offspring are exposed to a dietary restriction at a later stage of development at which the body plan is already at an advanced stage, necessitating a different manner of adaptive response: rapid reallocation of resources towards snout growth to enhance prey capture ability. However, this adaptive interpretation should be made with caution given that (1) snout length is not the only morphological parameter that would contribute to prey capture, and (2) prey capture ability is not equivalent to feeding performance. Indeed, while long snouts may improve prey capture ability by cranial rotation, shorter snouts attain better suction (Van Wassenbergh et al., 2013).

### DE of genes in newborn offspring

Newborn offspring in three out of four treatment groups exhibited gene expression changes relative to MW newborns to some extent, but EP newborns exhibited the most substantial changes in gene expression. This is also not surprising considering that the paternal dietary manipulation was the most recently enacted, and, as evidenced by the morphological changes on day 10, these newborns were actively undergoing a different trajectory of morphological development. The degree of differential expression across several differentially expressed genes correlated with the chronological order of the dietary treatments, further indicating that susceptibility to environmentally induced gene dysregulation increases with time during the reproductive process.

Interestingly, among downregulated genes in the EP group were those associated with fatty acid metabolism (fatty acid binding proteins 2 and 10-A, phospholipase A2), suggestive of molecular pathways underlying an adaptive response towards differential fatty acid utilisation. Several heat shock protein (HSP) family members were also differentially expressed and mostly downregulated. As PUFAs have been previously demonstrated to enhance the production of HSPs under stress conditions (Brand et al., 2010; Samples et al., 1999), it is possible that reduced PUFA availability may have been restrictive to HSP production in EP offspring. Although little to no differential expression was detected in treatment groups other than EP, the MC and PC samples nevertheless formed distinct clusters on the PCA plot. This suggests that there may have been a greater degree of treatment-induced alterations to gene expression but there was insufficient power to detect them. Despite the morphological alterations in newborns from the BC treatment, we detected no significant alterations to gene expression in offspring from this treatment. However, this is not surprising as, in contrast to EP offspring, the developmental alterations which appeared to be induced by the BC treatment had already taken place by the time of parturition. It also suggests that despite being smaller, BC newborns were not subject to substantial dysregulation in their physiology, further suggestive of an adaptive response to maintain normal physiological functions in the face of growth-restrictive conditions.

Because of a low level of read mapping and the use of whole-organism samples, the changes we could detect were probably restricted either to highly DE genes or to differential expression occurring simultaneously in many parts of the organism. This would explain why the only significantly DE pathway was a ribosomal protein pathway, as transcriptional regulation is a fundamental process which occurs throughout the whole organism. Ribosomal protein pathway downregulation may be indicative of a state of increased cellular stress (Hayashi et al., 2014), which can be induced by nutrient deficiency, as ribosomal biogenesis consumes >60% of cellular energy (Zhou et al., 2015). It would be expected that a state of stress – either nutritional stress or, for instance, hypoxia resulting from impairment of gas exchange functions in the pouch – would be induced in EP offspring at the time of manipulation, especially given the higher energetic demands of more advanced-stage embryos.

### Limitations and future work

This study implemented a complex experimental framework to address questions regarding a reproductive system of which many aspects remain elusive. Consequently, a number of key limitations and areas for future work must be acknowledged.

Firstly, our examination of a broad range of time windows was at the expense of sample size, which presumably limited our statistical power such that we were only able to detect the most prominent responses to paternal diet.

Secondly, although allowing couples to produce a variable number of broods prior to dietary manipulation allowed us to complete the experiment within a single continuous time period (thus avoiding the need to carry out the experiment in batches), we concede that variation in the number of broods that couples produced may have introduced additive effects that influenced the results; for instance, if maternal investment increases over successive courtships and mating events with the same male. However, any confounding influence of successive broods would have been mitigated as a result of (1) all experimental broods having been collected within the same 11 week period and (2) the number of broods produced by the couples prior to paternal dietary manipulation being approximately evenly distributed across the treatment groups. We also note that, as couples derived from mixed sex stock, it is possible that many of the individuals had already mated prior to the study and therefore unavoidably producing this variation regardless of the study design.

Thirdly, we did not directly compare the effects of commercial diet during specific time windows with the effects of commercial diet fed for the entirety of the experimental period (i.e. both preconception and throughout pregnancy). However, a similar manipulation was imposed on breeding male seahorses in our previous study (Otero-Ferrer et al., 2016), in which we found that offspring produced from males fed the commercial diet both before conception and throughout pregnancy produced offspring with increased standard length but poorer survival compared with offspring of wild-fed males, possibly because the snout length was not increased to the same degree as standard length, thus impeding feeding ability. Interestingly, in the present study, the BC treatment reduced standard length but, similar to our previous study, left snout length unchanged. This suggests that the growth rate of the snout during embryonic development remains constant while the growth of other size traits can be perturbed in different ways in response to paternal diet. However, the finding that BC and MC treatments in the present study induced opposite effects on standard length to the sustained commercial diet treatment in our previous study is puzzling and thus further study is needed to explain this disparity.

Finally, although we have shown that seahorse reproduction is sensitive to paternal PUFA availability during specific time periods, we did not address the specific mechanisms that may explain this sensitivity. Although it is plausible that early (pre-hatching) embryos respond directly to paternally derived nutrients, especially given that nutrients can be transferred from father to developing offspring in the brood pouch of pipefish (Haresign and Shumway, 1981; Ripley and Foran, 2009), it has nevertheless not been established whether PUFAs, which are large molecules, are able to cross through the chorion-like envelope enclosing the seahorse embryo in early development (Novelli et al., 2018). It may be more likely that developmental changes result from differential resource utilisation to counteract stress imposed by impairments to osmoregulatory or gas exchange functions of the pouch (Ripley and Foran, 2009; Stölting and Wilson, 2007), or, additionally, in the case of BC treatment, differential maternal investment in response to a perceived reduction in male quality. In both of these cases, smaller size traits in BC and MC offspring would simply be the result of a lack of available resources at a time when they would normally be recruited for growth. Thus, the relative contributions of these different hypothesised mechanisms should be addressed in future studies. The fitness consequences of the observed dietary effects could also be further explored – for instance, whether the enlarged snouts in EP offspring improve their foraging ability.

### Conclusions

We have demonstrated the existence of critical windows of environmental sensitivity in a seahorse model of male pregnancy, showing that paternal diet has different influences on the offspring depending on the timing of manipulation, at both phenotype and gene expression levels. In contrast to mammalian pregnancy, the outcome of seahorse pregnancy is especially sensitive to manipulation at the end of the pregnancy, reflective of the embryos being mostly dependent on maternal nutrition until later stages of development. Furthermore, the outcome of a given pregnancy also appears to be especially sensitive to sub-optimal diet encountered during the previous pregnancy, reflecting either deterioration of the regulatory functions of the brood pouch or reduced maternal investment in yolk resources in response to reduced perceived male quality, or a combination thereof. Our results provide evidence that the paternal physiological environment is adaptively important in relation to the fitness of progeny in seahorses, suggesting that the sophistication of the male seahorse brood pouch has permitted the evolution of a set of intricate functions analogous to maternal–embryo communication in mammalian pregnancy, but that the reliance of early embryos on yolk-derived nutrients means that the windows of sensitivity necessarily differ between mammals and syngnathids.

## Supplementary Material

Supplementary information
