# Supplementary material for: Time-critical influences of gestational diet in a seahorse model of male pregnancy
Source: J Exp Biol. 2020 Feb 7;223(3):jeb210302. doi: 10.1242/jeb.210302 (PMC7033721; doi:10.1242/jeb.210302)
Supplement: Supplementary information [file jexbio-223-210302-s1.pdf]

Table S1. Dates of parturition and brood sizes both for the experimental broods examined in response to paternal dietary manipulation, and the respective previous broods. Inter-brood interval (time between previous parturition and parturition of experimental brood) and the number of the brood that was tested (i.e. experimental brood plus all previous untested broods) are also shown.

Tank	Treatment	Prev. brood		Exp. Brood		Time interbrood (d)	Brood tested
		Date born	Brood size	Date born	Brood size		
B27	MW	23/03/2014	166	05/04/2014	173	15	6
B17	MW	10/02/2014	298	25/02/2014	485	14	2
R1	MW	22/02/2014	111	10/03/2014	316	16	2
R5bis	MW	22/02/2014	366	08/03/2014	349	14	4
R6	MW	18/02/2014	245	05/03/2014	316	15	5
B29	BC	09/04/2014	205	22/04/2014	135	13	7
R7	BC	16/03/2014	277	29/03/2014	284	13	5
B110	BC	19/03/2014	395	01/04/2014	360	13	7
R10bis	BC	27/04/2014	150	11/05/2014	148	14	5
B28	PC	01/05/2014	283	16/05/2014	236	15	7
B18	PC	08/03/2014	308	22/03/2014	358	14	5
R2	PC	08/03/2014	430	22/03/2014	562	14	4
B111	PC	21/03/2014	303	03/04/2014	468	13	3
R5	MC	08/03/2014	366	22/03/2014	457	14	5
R10	MC	02/03/2014	466	15/03/2014	583	13	6
B112	MC	22/04/2014	280	04/05/2014	283	12	2
B210	EP	27/04/2014	234	09/05/2014	290	12	4
B212	EP	18/02/2014	287	02/03/2014	470	13	4
R4	EP	06/04/2014	205	20/04/2014	356	14	2
R9	EP	13/04/2014	109	26/04/2014	200	13	2

Journal of Experimental Biology • Supplementary information

Table S2. Numbers of brood replicates and total numbers of individual offspring used for size measurements on day 0 (newborns) and day 10, respectively.

	D0		D10	
**Treatment**	**N broods**	**n offspring**	**N broods**	**n offspring**
MW	5	44	3	18
BC	4	41	2	12
PC	4	39	4	20
MC	3	30	2	12
EP	4	38	3	18

Table S3: differentially expressed genes at FDR ≤ 0.05 in commercial diet at the end of pregnancy (EP) *vs* wild-type diet throughout (MW) comparison. The differential expression analysis was conducted with edgeR. The table is sorted based on FDR values in the descending order.

RefSeq ID	Gene	Type	log_2FC	FDR
XM_019872513.1	ribosomal protein S14 rps14	coding	-1.61	6.31E-05
XM_019872752.1	ribosomal protein L17 rpl17	coding	-1.79	8.48E-05
XM_019875670.1	integrator complex subunit 6-like LOC109519254	coding	1.47	1.50E-04
XM_019880417.1	ribosomal protein L29 rpl29	coding	-1.94	2.70E-04
XR_002170586.1	uncharacterized LOC109527812 LOC109527812	ncRNA	2.63	3.67E-04
XM_019868925.1	hepcidin-like LOC109515263	coding	-2.3	6.46E-04
XM_019869045.1	ribosomal protein S9 rps9	coding	-1.45	6.46E-04
XM_019870925.1	RNA polymerase II subunit F polr2f	coding	-1.16	7.86E-04
XM_019876031.1	protein reprimo A-like LOC109519470	coding	-3.3	1.16E-03
XM_019891459.1	CREB-ATF bZIP transcription factor crebzf	coding	-1.23	1.30E-03
XM_019896332.1	ribosomal protein S15 rps15	coding	-1.35	1.32E-03
XM_019860549.1	ribosomal protein S23 rps23	coding	-1.28	1.84E-03
XM_019886437.1	family with sequence similarity 46 member B fam46b	coding	-1.27	2.81E-03
XM_019895011.1	FAU, ubiquitin like and ribosomal protein S30 fusion fau	coding	-1.48	2.96E-03
XM_019887735.1	4F2 cell-surface antigen heavy chain-like LOC109526434	coding	-1.53	3.00E-03
XM_019881723.1	myosin light chain 4-like LOC109522893	coding	-1.57	3.59E-03
XM_019858654.1	chymotrypsin A-like LOC109508622, partial	coding	-2.04	3.68E-03
XM_019871789.1	ribosomal protein L36 rpl36	coding	-1.18	3.81E-03
XM_019896330.1	ribosomal protein S28 rps28	coding	-1.21	3.83E-03
XM_019867928.1	40S ribosomal protein S27 LOC109514644	coding	-1.23	4.67E-03
XM_019894448.1	ribosomal protein L24 rpl24	coding	-1.02	4.67E-03
XM_019867578.1	ribosomal protein L12 rpl12	coding	-1.24	4.67E-03
XM_019895021.1	heat shock protein HSP 90-alpha-like LOC109531014	coding	1.14	4.82E-03
XM_019895768.1	ribosomal RNA processing 9, small subunit SSU processome component, homolog yeast rrp9	coding	-3.05	5.08E-03
XM_019856814.1	tropomyosin alpha-1 chain-like LOC109507404	coding	-1.31	5.08E-03
XM_019876398.1	dynein cytoplasmic 1 light intermediate chain 2 dync1li2	coding	1.04	5.08E-03
XM_019868745.1	putative uncharacterized protein DDB G0271974 LOC109515148, partial	coding	1.81	5.08E-03
XM_019877908.1	ankyrin repeat domain 9 ankrd9	coding	-1.71	5.21E-03
XM_019863587.1	ribosomal protein lateral stalk subunit P1 rplp1	coding	-1.14	5.21E-03
XM_019860636.1	gamma-crystallin M3-like LOC109510383	coding	-1.88	5.92E-03
XM_019889088.1	up-regulated during skeletal muscle growth 5 homolog mouse usmg5	coding	-1.33	6.37E-03
XM_019891754.1	cGMP-dependent protein kinase 1-like LOC109528789	coding	1.11	6.88E-03
XM_019895687.1	THAP domain-containing protein 2-like LOC109531395	coding	-1.21	6.88E-03
XM_019864193.1	beta,beta-carotene 9p,10p-oxygenase-like LOC109512427	coding	1.07	6.88E-03
XM_019863356.1	four and a half LIM domains 1 fhl1	coding	-1.8	7.01E-03
XM_019886125.1	basic transcription factor 3 btf3	coding	-1.03	7.01E-03
XM_019885986.1	eukaryotic translation elongation factor 1 delta eef1d	coding	-1.07	7.07E-03
XM_019893654.1	sodium- and chloride-dependent GABA transporter 2-like LOC109530091	coding	-1.83	7.22E-03
XM_019890963.1	heat shock protein family B small member 7 hspb7	coding	-2.08	7.30E-03
XM_019865608.1	ribosomal protein L38 rpl38	coding	-1.15	7.67E-03
XM_019883190.1	troponin I, slow skeletal muscle-like LOC109523783	coding	-1.09	7.67E-03
XM_019893709.1	ribosomal protein S16 rps16	coding	-1.27	7.90E-03
XM_019876848.1	gamma-crystallin M3-like LOC109519960	coding	-2.07	7.90E-03
XM_019874743.1	ring finger protein 20 rnf20	coding	0.88	8.10E-03
XM_019880124.1	ribosomal protein L14 rpl14	coding	-1.1	8.10E-03

Journal of Experimental Biology • Supplementary information

XM_019858587.1	regulator of G-protein signaling 9-like LOC109508571	coding	-1.21	8.10E-03
XM_019884653.1	natterin-3-like LOC109524650	coding	-1.38	8.76E-03
XM_019874268.1	lens fiber membrane intrinsic protein-like LOC109518450	coding	-1.51	8.98E-03
XM_019873361.1	heat shock protein beta-11-like LOC109517888	coding	-1.33	9.24E-03
XM_019896154.1	ribosomal protein S19 rps19	coding	-0.99	1.00E-02
XM_019888877.1	uncharacterized LOC109527143 LOC109527143	coding	1.03	1.00E-02
XM_019865580.1	guanine nucleotide-binding protein GI-GS-GO subunit gamma-T2-like LOC109513270	coding	-1.54	1.00E-02
XM_019889057.1	fibroblast growth factor 8 fgf8	coding	1.67	1.10E-02
XM_019891814.1	adhesion molecule with Ig like domain 2 amigo2	coding	1.45	1.10E-02
XM_019873000.1	betaine--homocysteine S-methyltransferase 1-like LOC109517690	coding	-1.61	1.10E-02
XM_019877622.1	ribosomal protein L28 rpl28	coding	-1.03	1.10E-02
XM_019882308.1	gastrula zinc finger protein XlCGF64.1-like LOC109523287	coding	1.57	1.12E-02
XM_019877669.1	ribosomal protein L37 rpl37	coding	-1.01	1.15E-02
XM_019860210.1	ribosomal protein S27a rps27a	coding	-0.97	1.15E-02
XM_019891194.1	dynein light chain Tctex-type 3-like LOC109528506	coding	-1.04	1.15E-02
XM_019858664.1	40S ribosomal protein S14 LOC109508636, partial	coding	-1.35	1.15E-02
XM_019867899.1	type-4 ice-structuring protein LS-12-like LOC109514623	coding	-1.13	1.19E-02
XM_019888887.1	retinol binding protein 1 rbp1	coding	-1.19	1.19E-02
XM_019888328.1	guanine nucleotide-binding protein Gi subunit alpha-1 LOC109526805	coding	1.06	1.29E-02
XM_019892468.1	retinal cone rhodopsin-sensitive cGMP 3p,5p-cyclic phosphodiesterase subunit gamma-like LOC109529209	coding	-1.71	1.36E-02
XM_019887858.1	synapse defective Rho GTPase homolog 1 syde1	coding	1.69	1.36E-02
XM_019856139.1	ribosomal protein L34 rpl34	coding	-1.08	1.38E-02
XM_019861552.1	exosome component 5 exosc5	coding	-1.45	1.40E-02
XM_019876857.1	gamma-crystallin M2-like LOC109519966	coding	-1.81	1.41E-02
XM_019891468.1	60S ribosomal protein L10 LOC109528618	coding	-1.01	1.41E-02
XM_019878911.1	desmoplakin dsp	coding	0.91	1.41E-02
XM_019893908.1	sarcospan sspn	coding	-1.05	1.41E-02
XM_019884254.1	eukaryotic translation initiation factor 4E binding protein 3 eif4ebp3	coding	-0.95	1.41E-02
XM_019881055.1	ribosomal protein S26 rps26	coding	-1.04	1.41E-02
XM_019894855.1	ribosomal protein L27a rpl27a	coding	-1	1.41E-02
XM_019895746.1	peptidyl-prolyl cis-trans isomerase FKBP5-like LOC109531447	coding	-1.06	1.41E-02
XM_019870822.1	ribosomal protein L11 rpl11	coding	-0.95	1.41E-02
XM_019892249.1	ribosomal protein L26 like 1 rpl26l1	coding	-1.03	1.41E-02
XM_019884949.1	eukaryotic translation initiation factor 2B subunit beta eif2b2	coding	-1.23	1.41E-02
XM_019857085.1	galactokinase 2 galk2	coding	-1.23	1.41E-02
XR_002167555.1	uncharacterized LOC109512579 LOC109512579	ncRNA	-2.49	1.46E-02
XR_002167900.1	uncharacterized LOC109513936 LOC109513936	ncRNA	-1.92	1.50E-02
XM_019888626.1	hypermethylated in cancer 2 protein-like LOC109526986	coding	1.05	1.50E-02
XM_019875311.1	ubiquitin A-52 residue ribosomal protein fusion product 1 uba52	coding	-1.02	1.50E-02
XM_019893931.1	complement C1q tumor necrosis factor-related protein 4-like LOC109530316	coding	-2.41	1.50E-02
XM_019893190.1	chymotrypsin A-like LOC109529759	coding	-1.85	1.50E-02
XM_019867182.1	cyclic nucleotide-gated cation channel beta-3-like LOC109514251	coding	-1.29	1.54E-02
XM_019884854.1	protein lin-7 homolog B LOC109524762	coding	-1.58	1.54E-02
XM_019893192.1	60S ribosomal protein L22-like 1 LOC109529757	coding	-1.04	1.58E-02
XM_019894032.1	phospholipase A2-like LOC109530375	coding	-1.73	1.58E-02
XM_019882679.1	solute carrier family 22 member 2-like LOC109523495	coding	-1.47	1.60E-02
XM_019868975.1	ribosomal protein L18 rpl18	coding	-1.49	1.67E-02
XM_019869327.1	gap junction gamma-1 protein-like LOC109515496	coding	1.09	1.70E-02
XM_019883566.1	RPE-retinal G protein-coupled receptor-like LOC109524001	coding	-1.56	1.72E-02

Journal of Experimental Biology • Supplementary information

XM_019888453.1	CCAAT-enhancer-binding protein beta-like LOC109526879	coding	-1.07	1.73E-02
XM_019868795.1	uncharacterized protein DDB G0271670-like LOC109515181, partial	coding	1.53	1.75E-02
XM_019894761.1	ribosomal protein S13 rps13	coding	-1.05	1.75E-02
XM_019892431.1	retinal cone rhodopsin-sensitive cGMP 3p,5p-cyclic phosphodiesterase subunit gamma-like LOC109529188	coding	-1.54	1.75E-02
XR_002169596.1	uncharacterized LOC109522676 LOC109522676	ncRNA	-0.9	1.77E-02
XM_019883965.1	matrix-remodeling-associated protein 5-like LOC109524255	coding	0.81	1.88E-02
XM_019883344.1	phosphodiesterase 6C pde6c	coding	-1.42	1.92E-02
XM_019873944.1	40S ribosomal protein S17 LOC109518228	coding	-0.88	1.92E-02
XM_019888271.1	metallothionein B LOC109526778	coding	-1.15	2.06E-02
XM_019868740.1	WAS-WASL-interacting protein family member 2-like LOC109515143	coding	-1.77	2.06E-02
XM_019895015.1	CD164 molecule cd164	coding	1.58	2.06E-02
XM_019868181.1	uridine phosphorylase 2 upp2	coding	-1.46	2.06E-02
XM_019863810.1	tropomodulin 4 tmod4	coding	-1.12	2.06E-02
XM_019873199.1	immunoglobulin superfamily member 11-like LOC109517811	coding	-3.46	2.06E-02
XM_019869638.1	NHP2 ribonucleoprotein nhp2	coding	-1.24	2.06E-02
XM_019857602.1	ATP synthase, H+ transporting, mitochondrial Fo complex subunit C3 subunit 9 atp5g3	coding	-1.24	2.14E-02
XM_019872121.1	GTPase activating Rap-RanGAP domain like 3 garnl3	coding	1.63	2.15E-02
XM_019894045.1	phospholipase A2, minor isoenzyme-like LOC109530383	coding	-1.4	2.15E-02
XM_019890197.1	crystallin beta B1 crybb1	coding	-1.57	2.19E-02
XM_019858686.1	Purkinje cell protein 4-like LOC109508675, partial	coding	-1.23	2.30E-02
XM_019886374.1	heat shock protein family B small member 6 hspb6	coding	-1.24	2.41E-02
XM_019882402.1	RAB, member of RAS oncogene family like 3 rabl3	coding	-1.04	2.41E-02
XM_019876560.1	collagen alpha-1XXVIII chain-like LOC109519786	coding	1.44	2.44E-02
XR_002166653.1	uncharacterized LOC109508400 LOC109508400	ncRNA	1.11	2.49E-02
XM_019874498.1	Golgi apparatus membrane protein TVP23 homolog B-like LOC109518569	coding	-1.2	2.50E-02
XM_019860497.1	HGH1 homolog hgh1	coding	-0.83	2.54E-02
XM_019888129.1	partner of NOB1 homolog pno1	coding	-0.78	2.65E-02
XM_019881376.1	musculoskeletal, embryonic nuclear protein 1 mustn1	coding	-1.39	2.65E-02
XM_019858381.1	ribosomal protein lateral stalk subunit P2 rplp2	coding	-0.96	2.70E-02
XM_019870832.1	adenylate kinase 2 ak2	coding	-0.95	2.83E-02
XM_019863532.1	homeobox A2 hoxa2	coding	2.5	2.83E-02
XM_019860928.1	ribosomal protein L35 rpl35	coding	-0.9	2.83E-02
XM_019856592.1	jagunal homolog 1 jagn1	coding	-1.04	3.03E-02
XM_019860483.1	cyclin D1 binding protein 1 ccndbp1	coding	-1.83	3.03E-02
XM_019886066.1	beta-crystallin B1-like LOC109525517	coding	-1.23	3.03E-02
XM_019857310.1	ribosomal protein L15 rpl15	coding	-0.86	3.03E-02
XR_002170583.1	uncharacterized LOC109527786 LOC109527786	ncRNA	-1.23	3.03E-02
XM_019892931.1	methyltransferase like 17 mettl17	coding	-1.05	3.05E-02
XM_019867114.1	LMBR1 domain-containing protein 2-B-like LOC109514218	coding	-1.84	3.08E-02
XM_019860823.1	solute carrier family 16 member 4 slc16a4	coding	-1.27	3.15E-02
XM_019874339.1	diphthamide biosynthesis 1 dph1	coding	-1.08	3.15E-02
XM_019861357.1	alpha-2-macroglobulin-like protein 1 LOC109510782	coding	2.2	3.23E-02
XM_019864167.1	glucose-6-phosphatase catalytic subunit g6pc	coding	-0.95	3.23E-02
XM_019867201.1	huntingtin interacting protein K hypk	coding	-0.8	3.23E-02
XR_002167735.1	uncharacterized LOC109513353 LOC109513353	ncRNA	1.73	3.34E-02
XM_019892096.1	claudin 12 cldn12	coding	0.94	3.34E-02
XM_019872293.1	cytochrome c oxidase assembly protein COX16 homolog, mitochondrial LOC109517258	coding	-1.1	3.36E-02
XM_019874731.1	ribosomal protein L39 rpl39	coding	-0.91	3.36E-02
XM_019858641.1	pyruvate dehydrogenase acetyl-transferring kinase isozyme 2, mitochondrial-like LOC109508605	coding	-1.35	3.36E-02
XM_019894643.1	family with sequence similarity 102 member A fam102a	coding	0.97	3.39E-02

Journal of Experimental Biology • Supplementary information

XM_019878719.1	Src homology 2 domain containing E she	coding	1.06	3.43E-02
XM_019894424.1	REV1, DNA directed polymerase rev1	coding	0.97	3.44E-02
XM_019879867.1	arf-GAP with SH3 domain, ANK repeat and PH domain-containing protein 1-like LOC109521813	coding	2.52	3.44E-02
XR_002168295.1	uncharacterized LOC109516088 LOC109516088	ncRNA	-1.14	3.44E-02
XR_002170305.1	uncharacterized LOC109526298 LOC109526298	ncRNA	-1.42	3.60E-02
XM_019870881.1	nectin-2-like LOC109516373	coding	-1.24	3.69E-02
XM_019889595.1	histidine-rich glycoprotein-like LOC109527582	coding	-1.78	3.69E-02
XM_019868320.1	NME-NM23 nucleoside diphosphate kinase 3 nme3	coding	-1.33	3.69E-02
XM_019894562.1	ATPase H+ transporting accessory protein 2 atp6ap2	coding	-0.86	3.69E-02
XM_019861806.1	immediate early response 2 ier2	coding	-2.56	3.69E-02
XM_019868650.1	claudin-7-A-like LOC109515080	coding	-0.93	3.69E-02
XM_019889826.1	ribosomal protein L27 rpl27	coding	-0.87	3.69E-02
XM_019878785.1	40S ribosomal protein S27-like LOC109521121	coding	-1.01	3.69E-02
XM_019875278.1	SH3 domain GRB2 like endophilin interacting protein 1 sgip1	coding	2.41	3.69E-02
XM_019895763.1	alpha-enolase-like LOC109531463	coding	-1.01	3.69E-02
XM_019889150.1	chromosome unknown C19orf53 homolog cunh19orf53	coding	-0.89	3.69E-02
XM_019875276.1	profilin-2-like LOC109519026	coding	0.98	3.69E-02
XM_019881407.1	DEAD-box helicase 27 ddx27	coding	-1.15	3.69E-02
XM_019860634.1	gamma-crystallin M3-like LOC109510382	coding	-1.57	3.69E-02
XM_019869053.1	fatty acid-binding protein 10-A, liver basic-like LOC109515345	coding	-1.31	3.69E-02
XM_019861686.1	solute carrier family 22 member 7-like LOC109510967	coding	-1.85	3.70E-02
XM_019896148.1	trypsin-2 LOC109531699	coding	-1.2	3.80E-02
XM_019865750.1	uncharacterized LOC109513350 LOC109513350	coding	1.18	3.80E-02
XM_019886290.1	ribosomal protein L13a rpl13a	coding	-0.84	3.87E-02
XM_019866861.1	ribosomal protein L21 rpl21	coding	-0.89	4.02E-02
XM_019874652.1	gastrula zinc finger protein XlCGF57.1-like LOC109518647	coding	1.04	4.02E-02
XM_019881469.1	40S ribosomal protein S11-like LOC109522712	coding	-1.09	4.02E-02
XM_019892069.1	prostate-associated microseminoprotein-like LOC109528972	coding	-2.24	4.05E-02
XM_019890501.1	tyrosine-protein kinase SgK223-like LOC109528161	coding	0.97	4.12E-02
XM_019887842.1	ATPase family, AAA domain containing 1 atad1	coding	-1.05	4.12E-02
XM_019878603.1	mex-3 RNA binding family member A mex3a	coding	0.78	4.14E-02
XM_019863614.1	vacuolar protein sorting 25 homolog vps25	coding	-1.05	4.16E-02
XM_019870795.1	ribosomal protein S5 rps5	coding	-1.02	4.16E-02
XM_019855881.1	glypican-1-like LOC109506871	coding	0.88	4.18E-02
XM_019877424.1	fatty acid binding protein 2 fabp2	coding	-1.01	4.18E-02
XM_019893197.1	puratrophin-1-like LOC109529765	coding	1.39	4.18E-02
XM_019881091.1	forkhead box P4 foxp4	coding	1.05	4.18E-02
XM_019868940.1	uncharacterized LOC109515277 LOC109515277	coding	0.92	4.33E-02
XM_019863757.1	ELL associated factor 1 eaf1	coding	-1.73	4.43E-02
XM_019873260.1	solute carrier family 22 member 6-like LOC109517842	coding	-1.06	4.46E-02
XM_019865821.1	Rac GTPase activating protein 1 racgap1	coding	1.29	4.54E-02
XM_019884519.1	zinc finger protein OZF-like LOC109524554	coding	1.11	4.59E-02
XM_019882191.1	zinc finger protein 410-like LOC109523197	coding	1.24	4.59E-02
XM_019861889.1	ribosomal protein S3A rps3a	coding	-0.79	4.59E-02
XM_019895856.1	inner mitochondrial membrane peptidase subunit 1 immp1l	coding	-1.27	4.62E-02
XM_019857273.1	cytochrome c oxidase subunit 5A, mitochondrial LOC109507663	coding	-0.87	4.68E-02
XM_019871792.1	transmembrane and coiled-coil domains 1 tmco1	coding	-0.81	4.68E-02
XM_019884750.1	phosphate regulating endopeptidase homolog, X-linked phex	coding	-1.71	4.68E-02
XM_019888154.1	cannabinoid receptor interacting protein 1 cnrip1	coding	-1.99	4.76E-02
XM_019895353.1	microtubule-associated protein 2-like LOC109531203	coding	1.07	4.76E-02
XM_019881570.1	regulation of nuclear pre-coding domain-containing protein 2-like LOC109522781	coding	1.21	4.79E-02
XM_019866086.1	translocase of outer mitochondrial membrane 7 tomm7	coding	-1.12	4.83E-02

Journal of Experimental Biology • Supplementary information

XM_019896281.1	LIM domain containing 2 limd2	coding	0.83	4.83E-02
XM_019862961.1	solute carrier family 25 member 33 slc25a33	coding	-1.02	4.85E-02
XM_019883075.1	transmembrane protein 168 tmem168	coding	2.21	4.90E-02
XM_019887480.1	BCL2 like 12 bcl2l12	coding	1.47	4.90E-02
XM_019871423.1	shootin-1-like LOC109516684	coding	0.9	4.90E-02
XM_019867994.1	exosome component 2 exosc2	coding	-0.83	4.96E-02

Table S4: differentially expressed genes at FDR ≤ 0.05 in commercial diet throughout (MC) *vs* wild-type diet throughout (MW) comparison. The differential expression analysis was conducted with edgeR. The table is sorted based on FDR values in the descending order.

RefSeq ID	Gene	Type	log_2FC	FDR
XR_002170586.1	uncharacterized LOC109527812 LOC109527812	ncRNA	2.68	2.82E-03
XM_019872752.1	ribosomal protein L17 rpl17	coding	-1.76	2.82E-03
XM_019875670.1	integrator complex subunit 6-like LOC109519254	coding	1.42	2.82E-03
XM_019895763.1	alpha-enolase-like LOC109531463	coding	-1.78	2.82E-03
XM_019856814.1	tropomyosin alpha-1 chain-like LOC109507404	coding	-1.68	3.10E-03
XM_019872513.1	ribosomal protein S14 rps14	coding	-1.37	1.39E-02
XM_019879405.1	oxidative stress-induced growth inhibitor 2-like LOC109521536	coding	1.7	1.85E-02
XM_019892658.1	endonuclease domain-containing 1 protein-like LOC109529330	coding	-4.47	2.74E-02
XM_019892442.1	guanylyl cyclase-activating protein 2-like LOC109529196	coding	-1.41	2.75E-02
XM_019880417.1	ribosomal protein L29 rpl29	coding	-1.68	2.88E-02
XM_019894837.1	solute carrier family 23 member 1-like LOC109530886	coding	-1.59	2.96E-02
XM_019881723.1	myosin light chain 4-like LOC109522893	coding	-1.63	2.96E-02
XM_019868533.1	xin actin-binding repeat-containing protein 2-like LOC109515008	coding	-1.44	3.24E-02
XM_019895021.1	heat shock protein HSP 90-alpha-like LOC109531014	coding	1.13	4.08E-02
XR_002166653.1	uncharacterized LOC109508400 LOC109508400	ncRNA	1.31	4.60E-02
XM_019888271.1	metallothionein B LOC109526778	coding	-1.38	4.60E-02
XM_019888887.1	retinol binding protein 1 rbp1	coding	-1.31	4.60E-02
XM_019863587.1	ribosomal protein lateral stalk subunit P1 rplp1	coding	-1.14	4.60E-02
XM_019869045.1	ribosomal protein S9 rps9	coding	-1.24	4.60E-02
XM_019860801.1	DPH3 homolog LOC109510463	coding	-1.7	4.89E-02
XM_019881645.1	inner membrane mitochondrial protein immt	coding	-2.43	4.89E-02

Journal of Experimental Biology • Supplementary information

Table S5: Enriched pathways and corresponding genes. Zebrafish (*D. rerio)* pathways were used as there are no KEGG pathways available for any seahorse subspecies. Genes differentially expressed in the EP (commercial diet at the end of pregnancy) vs MW (wild-type diet throughout) comparison at FDR ≤ 0.1 were included in the analysis. The enrichment analysis was conducted with the gage() function from gage package in R with the parameter same.dir = TRUE. For every pathway represented in the table, the corresponding differentially expressed genes are listed in the following rows marked by an indent. Column "DE Genes" lists the number of differentially expressed genes at FDR ≤ 0.1 that belong to the corresponding pathway.

Pathway ID	*Pathway Name*	*DE genes*	*FDR*	
		**Gene name**	**log_2FC**	**FDR**
dre03010	***Ribosome***	***48***	***0.0009***	
		ribosomal protein L17 rpl17	-1.79	8.48E-05
		ribosomal protein L29 rpl29	-1.94	2.70E-04
		ribosomal protein S9 rps9	-1.45	6.46E-04
		ribosomal protein S15 rps15	-1.35	1.32E-03
		ribosomal protein S23 rps23	-1.28	1.84E-03
		FAU, ubiquitin like and ribosomal protein S30 fusion fau	-1.48	2.96E-03
		ribosomal protein L36 rpl36	-1.18	3.81E-03
		ribosomal protein S28 rps28	-1.21	3.83E-03
		40S ribosomal protein S27-like LOC109519444	-0.78	5.02E-02
		ribosomal protein L24 rpl24	-1.02	4.67E-03
		ribosomal protein L12 rpl12	-1.24	4.67E-03
		ribosomal protein lateral stalk subunit P1 rplp1	-1.14	5.21E-03
		ribosomal protein L38 rpl38	-1.15	7.67E-03
		ribosomal protein S16 rps16	-1.27	7.90E-03
		ribosomal protein L14 rpl14	-1.1	8.10E-03
		ribosomal protein S19 rps19	-0.99	1.00E-02
		ribosomal protein L28 rpl28	-1.03	1.10E-02
		ribosomal protein L37 rpl37	-1.01	1.15E-02
		ribosomal protein S27a rps27a	-0.97	1.15E-02
		40S ribosomal protein S14 LOC109508636, partial	-1.35	1.15E-02
		ribosomal protein L34 rpl34	-1.08	1.38E-02
		60S ribosomal protein L10 LOC109528618	-1.01	1.41E-02
		ribosomal protein S26 rps26	-1.04	1.41E-02
		ribosomal protein L27a rpl27a	-1	1.41E-02
		ribosomal protein L11 rpl11	-0.95	1.41E-02
		ribosomal protein L26 like 1 rpl26l1	-1.03	1.41E-02
		ubiquitin A-52 residue ribosomal protein fusion product 1 uba52	-1.02	1.50E-02
		60S ribosomal protein L22-like 1 LOC109529757	-1.04	1.58E-02
		ribosomal protein L18 rpl18	-1.49	1.67E-02
		ribosomal protein S13 rps13	-1.05	1.75E-02
		40S ribosomal protein S17 LOC109518228	-0.88	1.92E-02
		ribosomal protein lateral stalk subunit P2 rplp2	-0.96	2.70E-02
		ribosomal protein L35 rpl35	-0.9	2.83E-02
		ribosomal protein L15 rpl15	-0.86	3.03E-02
		ribosomal protein L39 rpl39	-0.91	3.36E-02

Journal of Experimental Biology • Supplementary information

		ribosomal protein L27 rpl27		-0.87	3.69E-02
		40S ribosomal protein S27-like LOC109521121		-1.01	3.69E-02
		ribosomal protein L13a rpl13a		-0.84	3.87E-02
		ribosomal protein L21 rpl21		-0.89	4.02E-02
		40S ribosomal protein S11-like LOC109507142		-1.02	6.55E-02
		ribosomal protein S5 rps5		-1.02	4.16E-02
		ribosomal protein S3A rps3a		-0.79	4.59E-02
		ribosomal protein S10 rps10		-0.8	5.64E-02
		60S ribosomal protein L22 LOC109528365		-0.74	5.83E-02
		ribosomal protein S15a rps15a		-0.79	7.58E-02
		ribosomal protein L37a rpl37a		-0.76	8.35E-02
		ribosomal protein L35a rpl35a		-0.83	9.05E-02
		ribosomal protein L31 rpl31		-0.68	9.78E-02
dre01100	***Metabolic pathways***		***24***	***0.27***	
		RNA polymerase II subunit F polr2f		-1.16	7.86E-04
		betaine--homocysteine S-methyltransferase 1-like LOC109517690		-1.61	1.10E-02
		phospholipase A2, minor isoenzyme-like LOC109530383		-1.4	2.15E-02
		uridine phosphorylase 2 upp2		-1.46	2.06E-02
		ATP synthase, H+ transporting, mitochondrial Fo complex subunit C3 subunit 9 atp5g3		-1.24	2.14E-02
		adenylate kinase 2 ak2		-0.95	2.83E-02
		glucose-6-phosphatase catalytic subunit g6pc		-0.95	3.23E-02
		NME-NM23 nucleoside diphosphate kinase 3 nme3		-1.33	3.69E-02
		alpha-enolase-like LOC109531463		-1.01	3.69E-02
		cytochrome c oxidase subunit 5A, mitochondrial LOC109507663		-0.87	4.68E-02
		sphingosine kinase 1-like LOC109514836		-3.2	5.18E-02
		ATP synthase, H+ transporting, mitochondrial Fo complex subunit F6 atp5j		-1.05	6.25E-02
		proline dehydrogenase 1, mitochondrial-like LOC109526050		-0.8	6.64E-02
		ribose-phosphate pyrophosphokinase 1 LOC109515823		-1.77	7.50E-02
		diacylglycerol kinase zeta dgkz		1.4	7.80E-02
		ATP synthase, H+ transporting, mitochondrial Fo complex subunit G atp5l		-0.9	7.96E-02
		galactose-3-O-sulfotransferase 1 gal3st1		1.72	8.00E-02
		lipocalin-like LOC109517954		-0.73	8.12E-02
		malate dehydrogenase, cytoplasmic-like LOC109512633		-0.67	8.69E-02
		cytochrome c oxidase subunit 7C, mitochondrial LOC109520638		-0.81	8.76E-02
		alkaline ceramidase 2 acer2		-0.76	9.04E-02
		retinol dehydrogenase 10 rdh10		0.78	9.27E-02
		sialic acid synthase-like LOC109519931		1.1	9.41E-02
		dermatan sulfate epimerase dse		1.15	9.46E-02
dre03018	***RNA degradation***		***6***	***NA***	
		exosome component 5 exosc5		-1.45	1.40E-02
		alpha-enolase-like LOC109531463		-1.01	3.69E-02
		exosome component 2 exosc2		-0.83	4.96E-02
		LSM1 homolog, mRNA degradation associated lsm1		-1.06	5.83E-02

Journal of Experimental Biology • Supplementary information

		CCR4-NOT transcription complex subunit 6 cnot6		0.71	6.90E-02
		non-canonical polyA RNA polymerase PAPD7-like LOC109521940		0.79	8.21E-02
dre04141	***Protein processing in endoplasmic reticulum***		***6 NA***		
		heat shock protein HSP 90-alpha-like LOC109531014		1.14	4.82E-03
		SEC24 homolog A, COPII coat complex component sec24a		0.73	5.42E-02
		Sec61 translocon gamma subunit sec61g		-0.89	6.85E-02
		signal sequence receptor subunit 4 ssr4		-0.94	7.67E-02
		derlin 2 derl2		-0.95	8.15E-02
		TNF receptor-associated factor 2-like LOC109520405		1.47	9.78E-02
dre00190	***Oxidative phosphorylation***		***6 NA***		
		ATP synthase, H+ transporting, mitochondrial Fo complex subunit C3 subunit 9 atp5g3		-1.24	2.14E-02
		cytochrome c oxidase subunit 5A, mitochondrial LOC109507663		-0.87	4.68E-02
		ATP synthase, H+ transporting, mitochondrial Fo complex subunit F6 atp5j		-1.05	6.25E-02
		cytochrome c oxidase subunit 7A2, mitochondrial-like LOC109531728		-0.99	7.72E-02
		ATP synthase, H+ transporting, mitochondrial Fo complex subunit G atp5l		-0.9	7.96E-02
		cytochrome c oxidase subunit 7C, mitochondrial LOC109520638		-0.81	8.76E-02
dre00230	***Purine metabolism***		***6 NA***		
		RNA polymerase II subunit F polr2f		-1.16	7.86E-04
		phosphodiesterase 6C pde6c		-1.42	1.92E-02
		adenylate kinase 2 ak2		-0.95	2.83E-02
		NME-NM23 nucleoside diphosphate kinase 3 nme3		-1.33	3.69E-02
		ribose-phosphate pyrophosphokinase 1 LOC109515823		-1.77	7.50E-02
		phosphodiesterase 6G pde6g		-1.21	7.58E-02
dre04744	***Phototransduction***		***5 NA***		
		regulator of G-protein signaling 9-like LOC109508571		-1.21	8.10E-03
		guanine nucleotide-binding protein Gt subunit alpha-2-like LOC109511837		-1.04	6.64E-02
		guanine nucleotide-binding protein GT subunit gamma-T1-like LOC109527668		-0.95	7.50E-02
		phosphodiesterase 6G pde6g		-1.21	7.58E-02
		visinin-like LOC109515266		-1.02	9.98E-02
dre04260	***Cardiac muscle contraction***		***4 NA***		
		tropomyosin alpha-1 chain-like LOC109507404		-1.31	5.08E-03
		cytochrome c oxidase subunit 5A, mitochondrial LOC109507663		-0.87	4.68E-02
		cytochrome c oxidase subunit 7A2, mitochondrial-like LOC109531728		-0.99	7.72E-02
		cytochrome c oxidase subunit 7C, mitochondrial LOC109520638		-0.81	8.76E-02
dre04810	***Regulation of actin cytoskeleton***		***4 NA***		
		profilin-2-like LOC109519026		0.98	3.69E-02
		cholinergic receptor muscarinic 4 chrm4		1.57	6.37E-02

Journal of Experimental Biology • Supplementary information

		myosin regulatory light chain 2, skeletal muscle isoform type 2-like LOC109531589		-0.86	6.65E-02
		actin-like LOC109508673, partial		0.69	9.98E-02
dre01200	***Carbon metabolism***		***4 NA***		
		alpha-enolase-like LOC109531463		-1.01	3.69E-02
		ribose-phosphate pyrophosphokinase 1 LOC109515823		-1.77	7.50E-02
		malate dehydrogenase, cytoplasmic-like LOC109512633		-0.67	8.69E-02
		esterase D esd		-1.26	8.97E-02
dre03008	***Ribosome biogenesis in eukaryotes***		***3 NA***		
		NHP2 ribonucleoprotein nhp2		-1.24	2.06E-02
		SNU13 homolog, small nuclear ribonucleoprotein U4-U6.U5 snu13		-0.94	8.93E-02
		NOP10 ribonucleoprotein nop10		-0.87	9.78E-02
dre03013	***RNA transport***		***3 NA***		
		eukaryotic translation initiation factor 4E binding protein 3 eif4ebp3		-0.95	1.41E-02
		eukaryotic translation initiation factor 2B subunit beta eif2b2		-1.23	1.41E-02
		eukaryotic translation initiation factor 3 subunit F eif3f		-0.65	6.12E-02
dre04010	***MAPK signaling pathway***		***3 NA***		
		JunD proto-oncogene, AP-1 transcription factor subunit jund		-1	7.48E-02
		transforming growth factor beta 2 tgfb2		0.68	9.78E-02
		TNF receptor-associated factor 2-like LOC109520405		1.47	9.78E-02
dre04144	***Endocytosis***		***3 NA***		
		arf-GAP with SH3 domain, ANK repeat and PH domain-containing protein 1-like LOC109521813		2.52	3.44E-02
		vacuolar protein sorting 25 homolog vps25		-1.05	4.16E-02
		vacuolar protein sorting-associated protein 26B-like LOC109507981		0.69	9.98E-02
dre04145	***Phagosome***		***3 NA***		
		dynein cytoplasmic 1 light intermediate chain 2 dync1li2		1.04	5.08E-03
		Sec61 translocon gamma subunit sec61g		-0.89	6.85E-02
		actin-like LOC109508673, partial		0.69	9.98E-02
dre04530	***Tight junction***		***3 NA***		
		claudin-7-A-like LOC109515080		-0.93	3.69E-02
		cingulin-like protein 1 LOC109507489		0.71	7.58E-02
		actin-like LOC109508673, partial		0.69	9.98E-02
dre00240	***Pyrimidine metabolism***		***3 NA***		
		RNA polymerase II subunit F polr2f		-1.16	7.86E-04
		uridine phosphorylase 2 upp2		-1.46	2.06E-02
		NME-NM23 nucleoside diphosphate kinase 3 nme3		-1.33	3.69E-02
dre00564	***Glycerophospholipid metabolism***		***3 NA***		
		phospholipase A2, minor isoenzyme-like LOC109530383		-1.4	2.15E-02
		glycerol-3-phosphate dehydrogenase 1 gpd1		-0.82	5.18E-02
		diacylglycerol kinase zeta dgkz		1.4	7.80E-02
dre00600	***Sphingolipid metabolism***		***3 NA***		
		sphingosine kinase 1-like LOC109514836		-3.2	5.18E-02
		galactose-3-O-sulfotransferase 1 gal3st1		1.72	8.00E-02
		alkaline ceramidase 2 acer2		-0.76	9.04E-02

Journal of Experimental Biology • Supplementary information

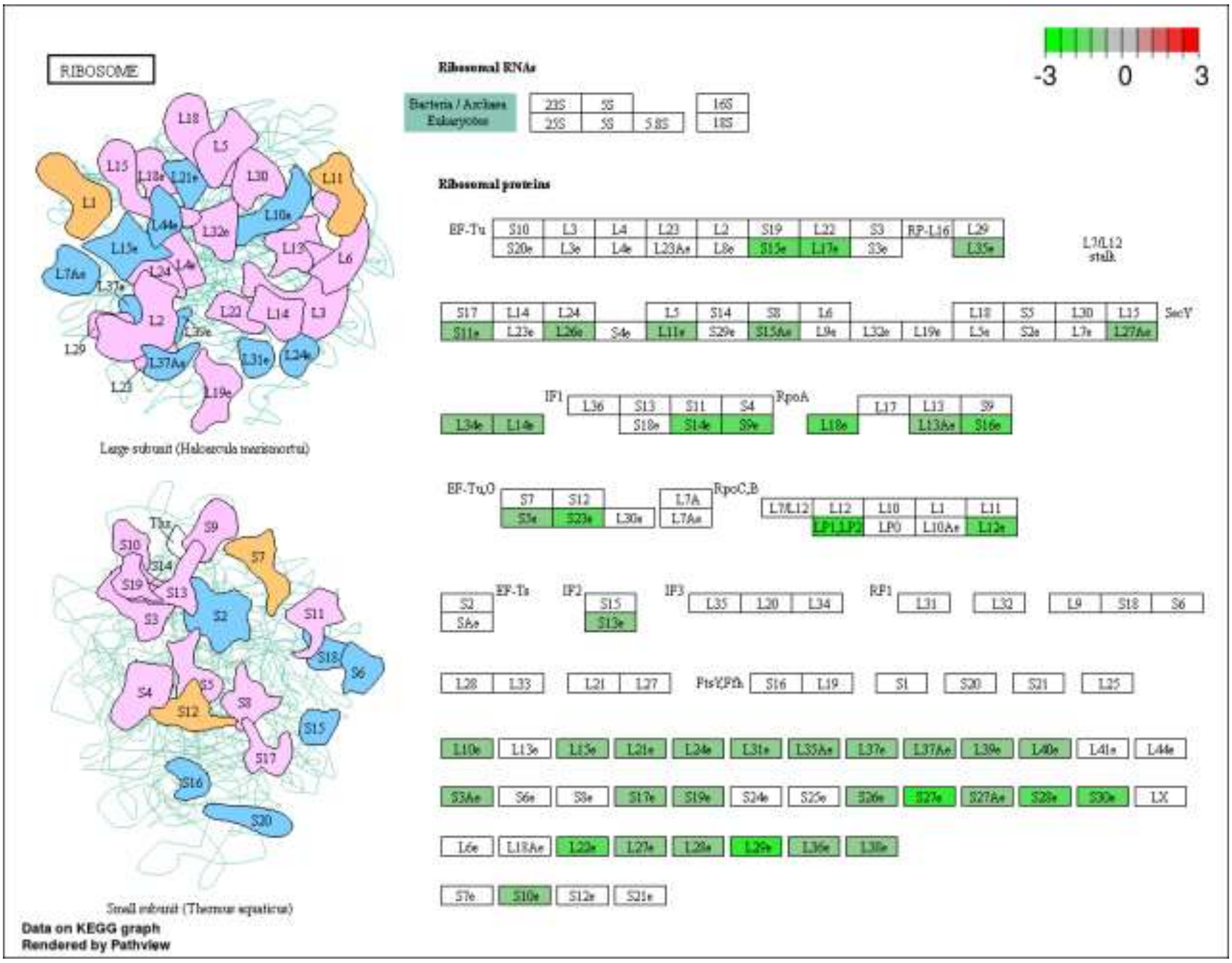

Figure S1: downregulated ribosomal protein genes in the EP vs MW comparison. This figure represents the ribosomal proteins, the expression of which is downregulated at the mRNA level (at FDR ≤ 0.1), based on the differential expression analysis between the EP (commercial diet at the end of pregnancy) and MW (wild-type diet throughout) groups. The log_2FC (log_2 of fold change) values are represented as a color code.
